# Predicting unconfined compressive strength of geopolymer-stabilized clays using a sector fruit fly–based extreme learning machine

**DOI:** 10.1038/s41598-026-47208-z

**Published:** 2026-04-17

**Authors:** Mohamed Abdellatief, Mohamed mortagi

**Affiliations:** 1 Department of Civil Engineering, Higher Future Institute of Engineering and Technology in Mansoura, Mansoura, Egypt; 2 Structural Engineering Department, Mansoura University, Mansoura, Egypt; 3 Faculty of Engineering, Mansoura National University, Gamasa, Egypt

**Keywords:** Geopolymer-stabilized soils, Clay soil, Extreme Learning Machine, Sector fruit fly optimization algorithm, Unconfined compressive strength, Engineering, Mathematics and computing

## Abstract

Accurate prediction of the unconfined compressive strength (UCS) of geopolymer-stabilized clayey soil is critical for geotechnical engineering. Conventional regression algorithms and even advanced machine learning approaches such as artificial neural networks often struggle to fully capture the highly non-linear interactions among soil properties and geopolymer mix parameters while maintaining computational efficiency and interpretability on limited datasets. To address these challenges, this investigation proposes a novel hybrid predictive framework based on a sector fruit fly optimization algorithm–enhanced extreme learning machine (SFOA-ELM) for improved UCS estimation. The model used 270 experimental records with 8 input variables (i.e., liquid limit, plasticity index, GGBS content, molarity). The baseline ELM achieved moderate performance (training R² = 0.9432, testing R² = 0.9054; RMSE = 1.55 MPa and 1.91 MPa, respectively). The predictive accuracy of the standard FOA-ELM improved to an R² of 0.9768 (training) and 0.9318 (testing), with RMSE values of 0.99 MPa (training) and 1.62 MPa (testing). The performance of the SFOA-ELM model improved to have excellent predictive performance with an R² value of 0.9775 (training) and 0.9446 (testing), and an RMSE value of 0.98 MPa (training) and 1.462 MPa (testing), respectively. The SFOA-ELM model reduced the testing MSE by 42% compared to ELM and by 19% compared to FOA-ELM. A SHAP analysis suggested that GGBS content, plasticity index and liquid limit were the most important for prediction. The statistical validations and residual analysis confirmed SFOA-ELM’s ability to generalize effectively and exhibit a tight distribution of errors. These findings show that the SFOA-ELM framework offers a robust, computationally efficient, and interpretable tool for geopolymer mix design and UCS prediction, providing practical decision-support for geotechnical engineering applications.

**Supplementary Information:**

The online version contains supplementary material available at 10.1038/s41598-026-47208-z.

## Introduction

Geopolymers are modern cementitious materials formed through the polymerization of aluminosilicates alongside alkaline activators. Ground granular blast furnace slag (GGBS), fly ash (FA), silica fume, metakaolin (MK), and glass powders are examples of industrial waste aluminosilicates that can be generated into geopolymers with alkaline activators^1,2^. The primary products from the geopolymerization process include calcium silicate hydrate (C–S–H), calcium aluminosilicate hydrate (C–A–S–H), and sodium aluminosilicate hydrate (N–A–S–H) and form a rigid three-dimensional tetrahedral framework^3–7^. The geometry of the tetrahedra allows for desirable characteristics such as high compressive strength, durability, shrinkage resistance; and moreover, responsible for almost no CO_2_ emissions from the reaction when compared to other binding materials^8–10^. In geotechnical engineering, Portland cement (PC) is the most commonly used binding material to improve the mechanical performance of soils and soil mixtures however this is concerning as PC has a huge environmental footprint and already generates significant CO₂ emissions^5,6^. Whereas there are bound to be benefits in technical performance with geopolymers with lower energy consumption, environmental footprint, and cost when compared to PC.

Previous studies on soil stabilization using geopolymers have revealed favorable findings^11–15^. Since the material possesses a dense microstructure and low shrinkage potential, Zhang et al.^14^ found that soil formation in clay with more than 8 wt% MK-based geopolymer decreased shrinkage strains compared to wt% PC. For unconfined compressive strength (UCS), there was potential with effective dosages of over 11 wt%; however, UCS increases between 7- and 28-day curing times were minor, which supports a rapid geopolymerization reaction. In another study, Zhang et al.^16^ reported that calcium-free MK-based geopolymers were able to stabilize sulfate-rich clay soils, such that swelling strains were noticeably lower than those stabilized with 4% lime. Further investigations have also demonstrated the role of precursor compositions in the soil stabilization systems. Khadka et al.^17^ and Aboulayt et al.^18^ investigated the MK–FA binary geopolymers for clay stabilization, noting that a 20% FA mixture achieved mechanical properties similar to pure MK, whereas FA contents above 40% diminished the compressive strength and elastic modulus due to reduced reactivity. Despite being widely used to stabilize soil, PC poses significant challenges for expansive and sulfate-rich clays due to ettringite-induced swelling, a high heat of hydration that leads to cracking, and vulnerability to sulfate attack, which can lower long-term strength and volume stability^19,20^. On the other hand, geopolymers have distinct technical advantages: they form a three-dimensional aluminosilicate network with less calcium, which decreases shrinkage, boosts resistance to acid environments and sulfate attack, enhances durability under wet-dry and freeze-thaw cycles, and successfully minimizes swelling in high-plasticity clays^5–7,10,21–25^. These properties make geopolymers particularly useful for stabilizing problematic expansive clays, where traditional cement often performs poorly or requires higher dosages at higher environmental costs. Additionally, Table 1 presents recent studies on soil stabilization using geopolymers with clay soil, demonstrating various precursors of geopolymers including volcanic ash (VA), GGBS, FA, MK and palm oil fuel ash (POFA) in combination with different alkali activators. The UCS of treated clay soils was measured from as low as 0.15 MPa to as high as 41.5 MPa depending on the premixed ratio of alkali binder, activator, precursor, and curing time.

It is known that the UCS of clay soils, particularly those stabilized with geopolymers, results from a number of factors. Extensive and time-consuming laboratory tests would have to be conducted in order to characterize a UCS typical of soil-stabilized geopolymers. Therefore, the predictive models are serve as an effective solution to this challenge. The existing models reported in previous literature rely on regression methods^26–32^ but are constrained by the assumption that there are preexisting relationships, whether linear or nonlinear, among the variables in the model. Machine learning (ML) methods have proven to be useful; these methods have emerged as valuable tools in various engineering fields^1,4–9,24,25,28–30,33,34^ that focus on predicting UCS of geopolymer-stabilized soils. Examples of these models include artificial neural networks (ANN)^35^, support vector machines (SVM)^36^, gradient boost (GB)^37^, multi-gene genetic programming (MGGP)^38^, and the more advanced hybrid neuro-fuzzy (NF)-group method of data handling (GMDH) with particle swarm optimization (PSO)^39^. For instance, Mozumder and Laskar^35^ generated an ANN model using a multilayer perceptron (MLP) with Bayesian regularization and obtained an R^2^ value of 0.9643 to predict the UCS. In this context, Mozumder et al.^40^ used SVM with an R^2^ of 0.9801; Soleimani et al.^41^ used MGGP to achieve an R^2^ of 0.9420 and mean absolute error (MAE) of 1.071 MPa; Javdanian and Lee^39^ used a NF-GMDH-PSO hybrid with R^2^ of 0.971, MAE of 0.231 MPa, RMSE of 0.401 MPa; Zeini et al.^42^ used RF to achieve an R^2^ of 0.9757 and RMSE of 0.9815 MPa. While these models have improved, ANN models and the results of the recent study of Mozumder et al.^35,40^ for geopolymer-stabilized clayey soils show good predictive accuracy but not usable equations for calculating results. While these models can assist in improving predictive accuracy, they are limited because of their small datasets, optimization inefficiencies, and lack of interpretable outputs, especially for clay soils with particular issues such as swelling and changing reactivity level.

**Table 1:** Summary of recent studies on soil stabilization employing geopolymers with clay soil.

Ref	Precursor type	Alkali activator	Alkali /binder ratio	UCS (MPa)	Curing time
^22^	VA	Sodium hydroxide (NH)	–	12.00	28d
^43^	VA in cement stabilized soil	NH	–	12.00	28d
^44^	VA/GGBS	NH	0.22	3.14	28d
^39^	FA/Slag	NH	0.85	24.26	28d
^45^	FA/Slag	NH + Na_2_SiO_3_	0.4	0.40	28d
^46^	FA/Slag	NH + Na_2_SiO_3_	0.65	11.3	28d
^17^	FA/MK with highly expansive soils	NH + Na_2_SiO_3_	–	–	–
^47^	GGBS	Na_2_SiO_3_	–	3.5	28d
^48^	GGBS	NH	0.85	24.26	28d
^49^	GGBS/BOFS with soft clay	CaO + MgO	0.3	7.41–8.44	90d
^13^	MK	KOH	–	0.85	28d
^50^	MK	NH	0.7	4.10	28d
^51^	Copper slag with clayey-sandy soil	NH + Na_2_SiO_3_	1	0.67	14d
^52^	POFA	NH + Na_2_SiO_3_	1.32	4.18	28d
^53^	POFA/glass fibers with sandy clay	KOH	–	5.7	28d
^53^	Recycled glass powder	NaOH	–	2.5	7d

The current study addresses the development and validation of a new prediction algorithm to estimate the UCS of geopolymer-stabilized clayey soils. To achieve this, extreme learning machine (ELM), fruit fly optimization algorithm (FOA)-based ELM, and an enhanced sector fruit fly optimization algorithm (SFOA)-based ELM (SFOA-ELM) were introduced to promote more efficient hyperparameter selection in ELM. In geotechnical engineering, ELM has recently emerged as a rapidly growing tool with the capacity to provide accurate predictions and serves as a complementary alternative to conventional modeling techniques. The FOA is a swarm-based intelligence algorithm that is inspired by the foraging behavior of fruit flies, which use both visual and olfactory senses to detect food^54^. The FOA begins by representing the candidate solutions to a problem as fruit flies randomly positioned in the solution space. The algorithm iteratively updates their positions to find better solutions. Unfortunately, the original FOA search is entirely random with fixed behavior, and radius, decisions that can severely reduce both the accuracy and stability. To overcome the challenges associated with the original FOA, the proposed SFOA introduced a sector-style search. Instead of being entirely random, the flies will distribute in a uniform manner along each of the search directions, while still maintaining a fixed step size by utilizing sine and cosine functions. This removes as much randomness as possible from the decisions made by the flies and higher stability and more efficient searching can lead to a higher convergence accuracy. Although the traditional machine learning models have demonstrated good predictive accuracy, they are frequently criticized for producing unintelligible results that offer little insight into the relative importance of input variables and their interactions, a crucial requirement in geotechnical engineering for mechanistic understanding and well-informed mix design. To address this limitation, the proposed SFOA-ELM framework is further combined with SHAP (SHapley Additive exPlanations) analysis to enhance model interpretability. Generally, the integrated SFOA-ELM–SHAP framework provides both accurate UCS predictions and transparent feature attribution, offering a robust, efficient, and interpretable decision-support tool for estimating the UCS of geopolymer-stabilized clayey soils.

## Methodology

### Study framework and data preparation

**Fig. 1:**
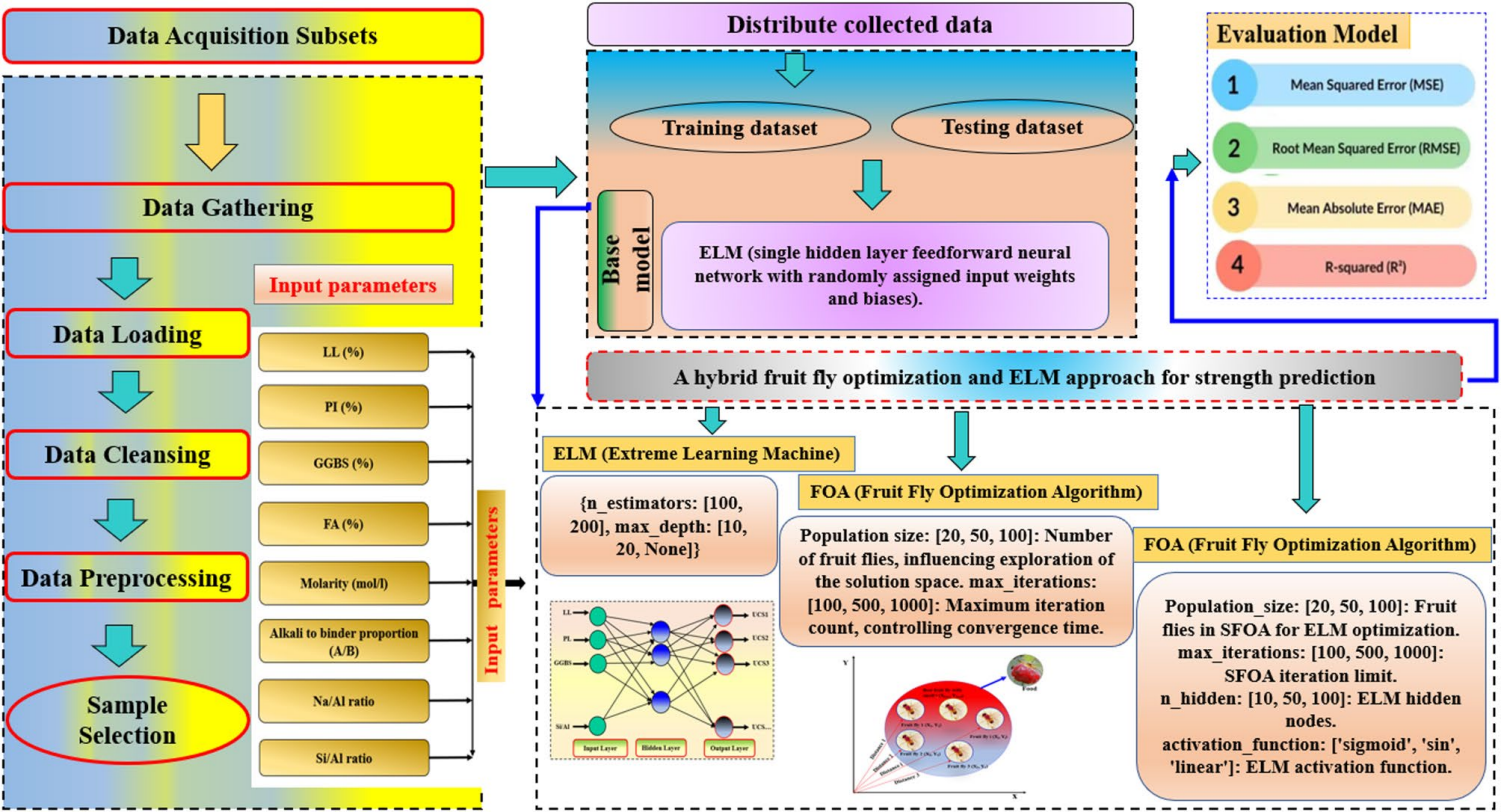
Framework of the current research.

**Fig. 2:**
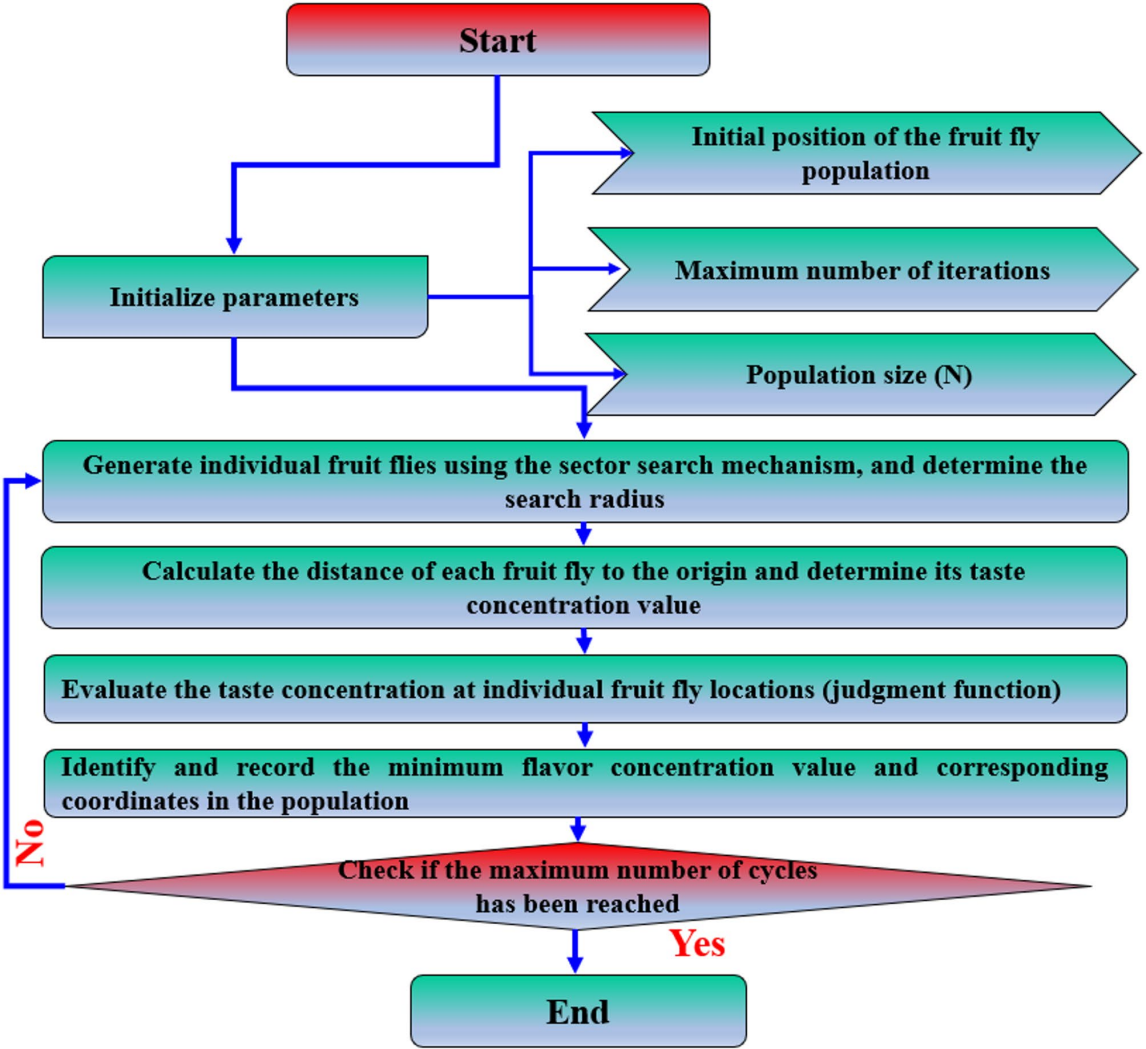
SFOA flowchart.

The primary objective of this research is to develop a robust and accurate prediction model for the UCS of cohesive soils stabilized with geopolymer using an ELM, FOA-ELM and SFOA-ELM approaches. The robustness of the models was additionally confirmed through SHAP values and statically tests. The overall research framework, including the proposed models, is displayed in Fig. 1. The database reported by Mozumder and Laskar^35^, which compiles UCS findings from several laboratory studies on geopolymer-stabilized clayey soils, provided the 270 experimental records used in this study. The dataset reflects the inherent heterogeneity of geopolymer stabilization studies by covering a broad range of precursor systems (GGBS, fly ash, and blended binders), activator concentrations, curing regimes, and clay types (low- to high-plasticity kaolinite-, illite-, and montmorillonite-based soils). Although the data were not generated under a single standardized protocol, introducing unavoidable inter-study variability in specimen preparation and testing, and exact record independence cannot be fully ensured, this dataset remains one of the largest publicly available compilations for UCS prediction of geopolymer-stabilized clays and has been widely adopted in previous machine learning benchmarks. The broad ranges of LL (37.7–116%), PI (14.1–88.5%), GGBS content (0–50%), and UCS (0–24.26 MPa) provide sufficient variability for developing and validating predictive models within this domain. This study uses 270 records, which are split into two phases: 80% of the data (216 records) is used for training phase and 20% is used for testing phase (54 records). As presented in Figs. 1 and 2 input variables were used to predict the UCS using the proposed ML models.

Table 2 provides the statistical summary of the dependent and independent parameters for the collected datasets. The input parameters utilized for this study were the liquid limit (LL, %) and plasticity index (PI, %) which provide measures of the properties of the cohesive soil. The fly ash (FA) and GGBS were the source materials for geopolymerization and form the component that was chemically bonded with the clayey soil. Within the geopolymer system, the parameters of interest were the alkali-to-binder ratios (A/B), the molar concentration of the alkali solution (Molarity, M), and the Si/Al and Na/Al atomic ratios. These inputs cover the main attributes of the key factors impacting the stabilization process. As illustrated in Table 2, the alkali solutions with different molarities 4 M, 8 M, 10 M, 12 M and 16 M were employed. The individual A/B ratios were 0.45, 0.65 and 0.85.

**Table 2:** The descriptive statistics of input features and output parameter for UCS.

Variables	Unit	Min	Mean	Std	Skewness	Max
LL	%	37.70	64.054	31.446	0.554	116
PI	%	14.07	44.598	29.844	0.553	88.46
GGBS	%	0	12.738	10.343	0.614	50
FA	%	0	1.663	4.466	3.135	20
Molarity	mol/l	4.00	11.996	3.466	-0.110	16
A/B	-	0.45	0.627	0.167	0.950	0.85
Na/Al	-	0.24	1.111	0.467	0.352	1.98
Si/Al	-	1.49	1.680	0.316	1.699	2.49
UCS	MPa	0	4.50	5.855	1.609	24.26

**Fig. 3:**
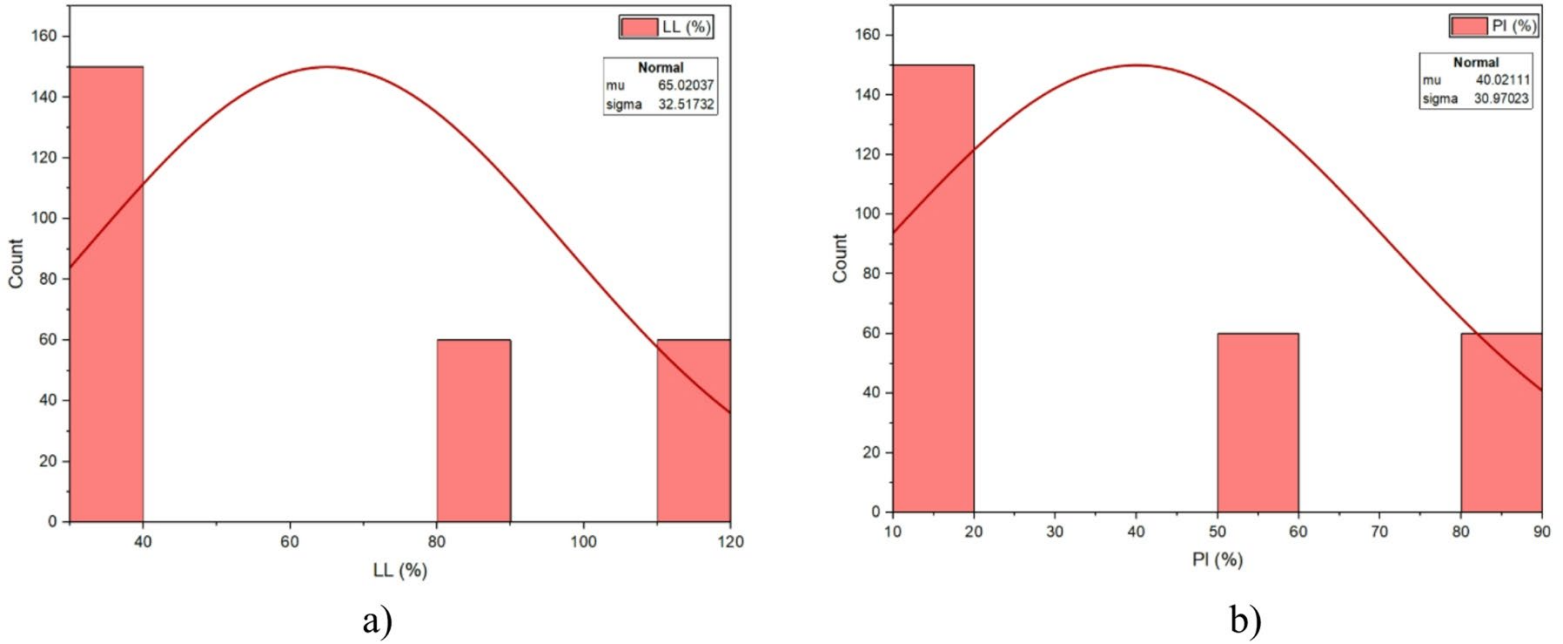
Histograms of soil properties **a**) LL and **b**) PI.

**Fig. 4:**
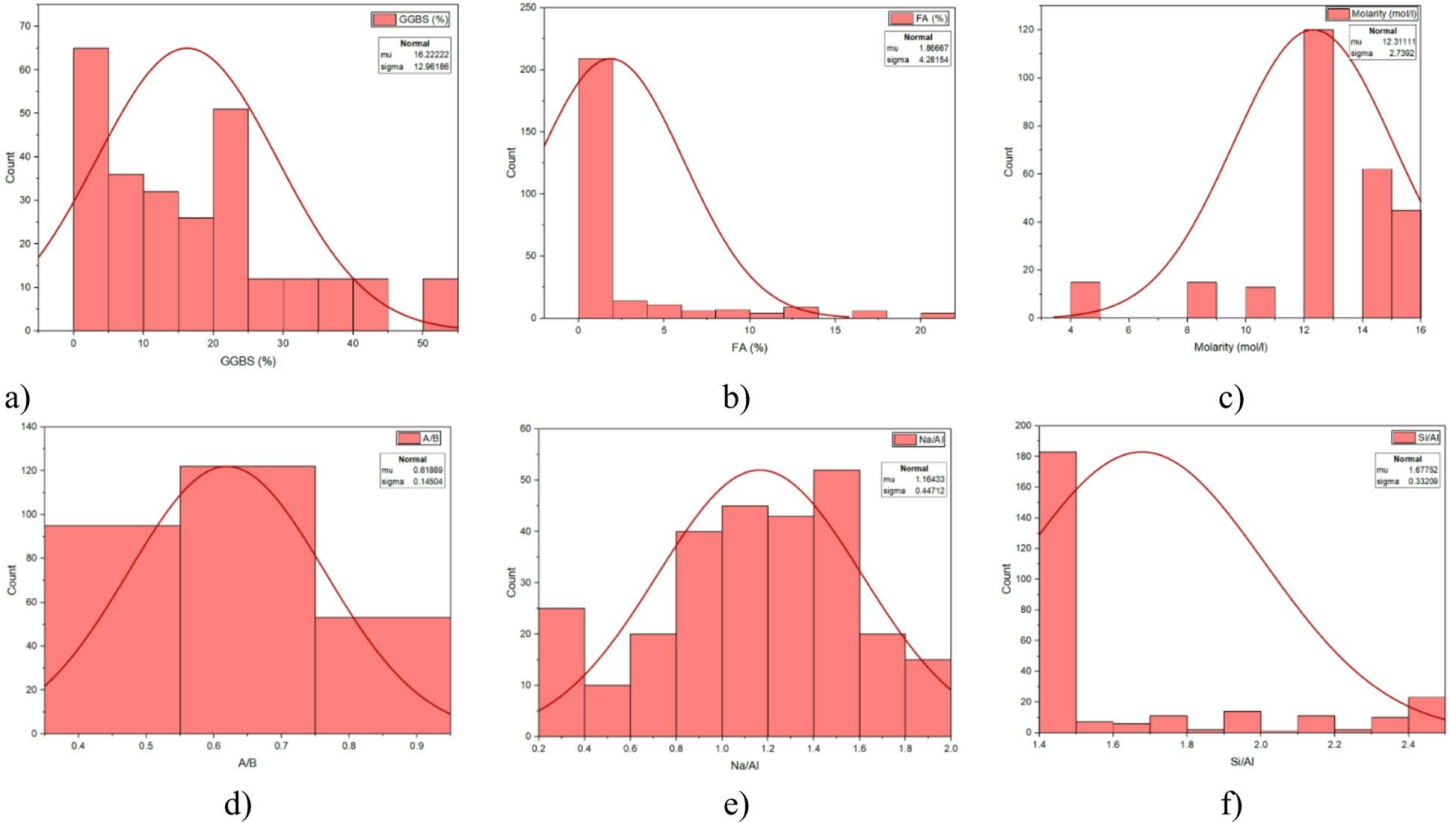
Frequency histograms of the input and output parameters **a**) GGBS, **b**) FA, **c**) Molarity, **d**) A/B, **e**) Na/Al, and **f**) Si/Al.

Figure 3 shows the frequency histograms for LL (%) and PI (%). The LL histogram (Fig. 3a) ranged from 37.70 to 116%, with a mean of 64.05% and a standard deviation of 31.45% with slight right skewness (skewness = 0.554) that is typical of natural clay-based material. The PI histogram (Fig. 3b) ranged from 14.07 to 88.46%; mean was 44.60% and standard deviation was 29.84% also with some mild skewness (0.55) indicating variability in soil plasticity and consistency limits throughout the 270 -sample set. Figure 4 outlines the assessments of primary parameters associated with geopolymerization across the dataset. The GGBS (Fig. 4a) ranged from 0% to 50%, with a mean of 12.22% and standard deviation of 12.01% demonstrating positive skewness (0.614) and also strongly peaking at lower percentages. The FA (Fig. 4b) ranged from 0% to 20%, with a mean of 1.86 and standard deviation of 2.81, showing extreme skewness (3.135) as it was dominated by low FA values. The molarity (Fig. 4c) ranged from 4 to 16 mol/l with a mean of 12.31 and standard deviation of 2.73, and resulted in a reasonably symmetric shape although there was a clear peak 12–14 mol/l. The A/B (Fig. 4d) ranged from 0.4 to 0.9, with a mean of 0.6180 and standard deviation of 0.145 and had a unimodal distribution with most measurements around 0.6–0.7. The Na/Al (Fig. 4e) ranged from 0.4 to 2.0, with a mean setting on 1.16 and standard deviation of 0.44, in line with a balanced distribution with a peak measured close to 1.0-1.2. The Si/Al (Fig. 4f) ranged from 1.6 to 2.4 with a mean at 1.67 and standard deviation of 0.33 and exhibited slight right skewness while there was measures clustering around 1.6–1.8, broadly reflective of the binder chemistry influence.

**Fig. 5:**
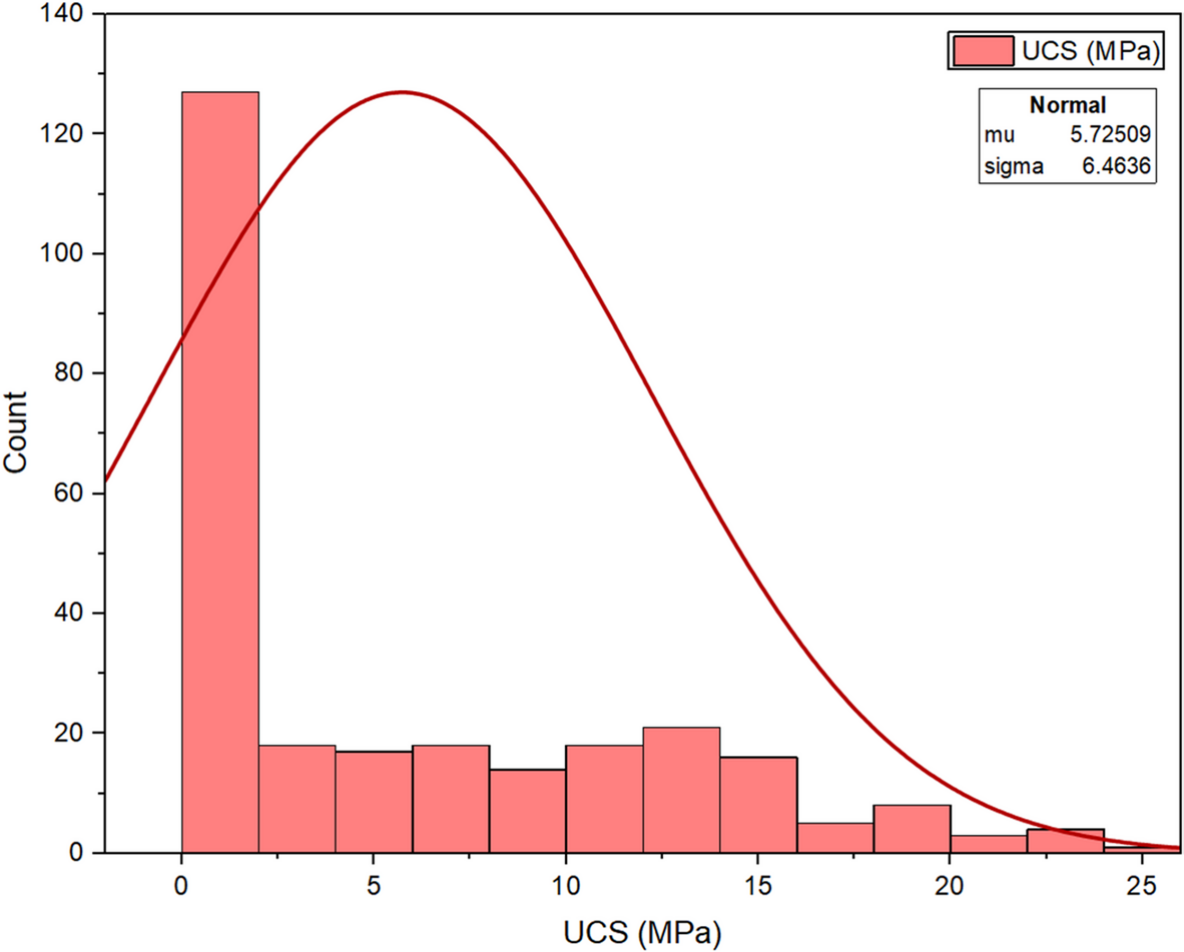
Output Variable (UCS).

Figure 5 presents the frequency histogram of UCS for the 270 soil samples. The UCS values range from 0 MPa to 24.26 MPa, with the average (mean) value at 4.50 MPa and a standard deviation of 5.86 MPa. The distribution of UCS illustrates a significant right-skewness (skewness of 1.60), with a peak occurring at approximately 5–10 MPa, reflecting the variability of strength based on input parameters such as binder weight and alkali ratios. The normal curve displayed provides some assistance with the assessment of the deviation from normality and illustrates how mix design characteristics can impact mechanical performance.

**Fig. 6:**
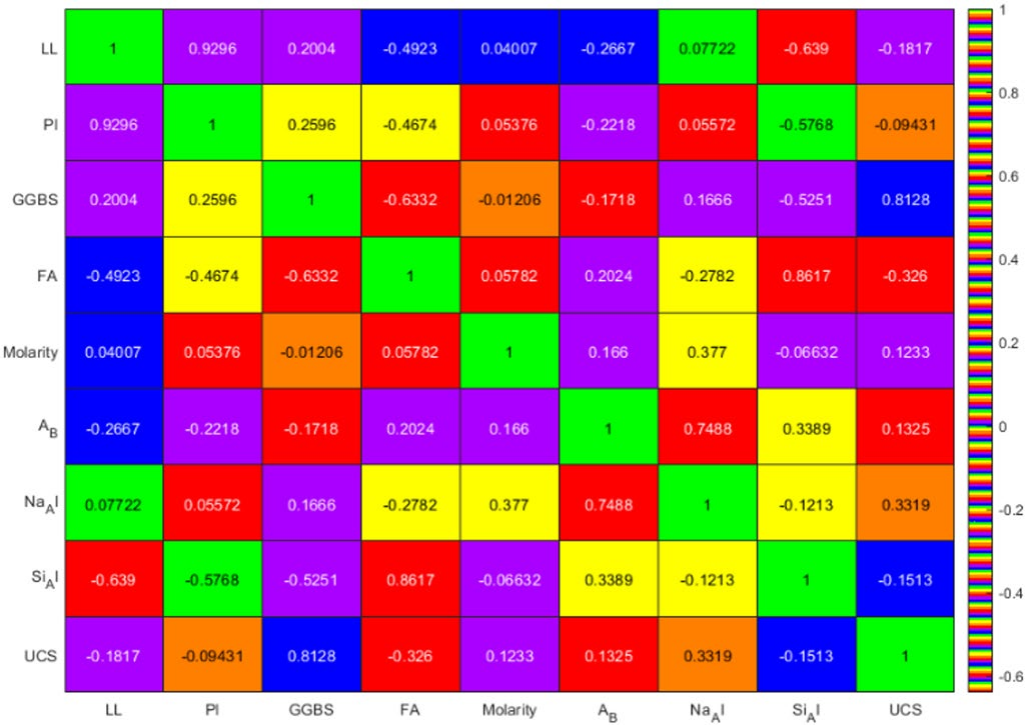
Correlation coefficient between features.

The Pearson correlation coefficients among the input parameters (LL, PI, GGBS, FA, molarity, A/B, Na/Al and Si/Al) and the output parameter (UCS) are shown in Fig. 6. The correlation coefficients range from − 1 to + 1, reflecting both the strength and direction of the relationships. A value of 0 indicates no correlation, and ± 1 indicates perfect correlation. Several key points can be made with this analysis. There is a very strong positive correlation with UCS and GGBS content (*r* = 0.818) and confirms its significance in enhancing UCS via pozzolanic reactivity within the geopolymer matrix. There is a somewhat moderate positive correlation with molarity (*r* = 0.123), indicating higher alkali solution concentration improves strength by a very small degree, consistent with better activation of the aluminosilicate species found in soil. The Na/Al ratio shows a moderate positive correlation (*r* = 0.319) and indicates that sodium ion ratio to aluminum ion is paramount to ensure effective geopolymerization while Si/Al ratio shows a weak negative correlation (*r* = − 0.070) which is indicative of little impact to UCS.

Amongst the soil parameters, LL and PI are highly associated (*r* = 0.688), which reflects the natural relationships between LL and PI in cohesive soils. GGBS and FA showed a weak positive relationship with (*r* = 0.280) but each can all be independently optimized for their relative contribution to strength. The A/B ratio showed a strong relationship with Na/Al (*r* = 0.748) showing the importance of meeting alkali balance in equilibrating the binder system. In contrast FA (*r* =–0.326), and PI (*r*=–0.543) showed moderate negative relationships with UCS, implying excessive FA or highly plastic soils would reduce strength from dilution or alter the cure. The other parameters showed weak relationships with UCS, A/B (*r* = 0.125) and Si/Al (*r* =–0.070) were weak, indicating either secondary effects or dependent on context. Low inter-parameter relationships (for example, molarity and Na/Al, *r* = 0.156) indicate a low inter-collinearity between parameters which additionally supports the assumption that approximately each input is statistically independent. Generally, the metrics highlight GGBS content and Na/Al ratio as potentially the most influential predictors of UCS with secondary influences from molarity and negative influences from FA and PI. However, the degree of variability and complex patterns of relationships suggests the system of geopolymer is complex and justifying the implementation of ML approaches such as ELM and optimized variants as they have the potential to better capture the complexity and shape of the data dispersion.

### ML models

#### ELM

**Fig. 7:**
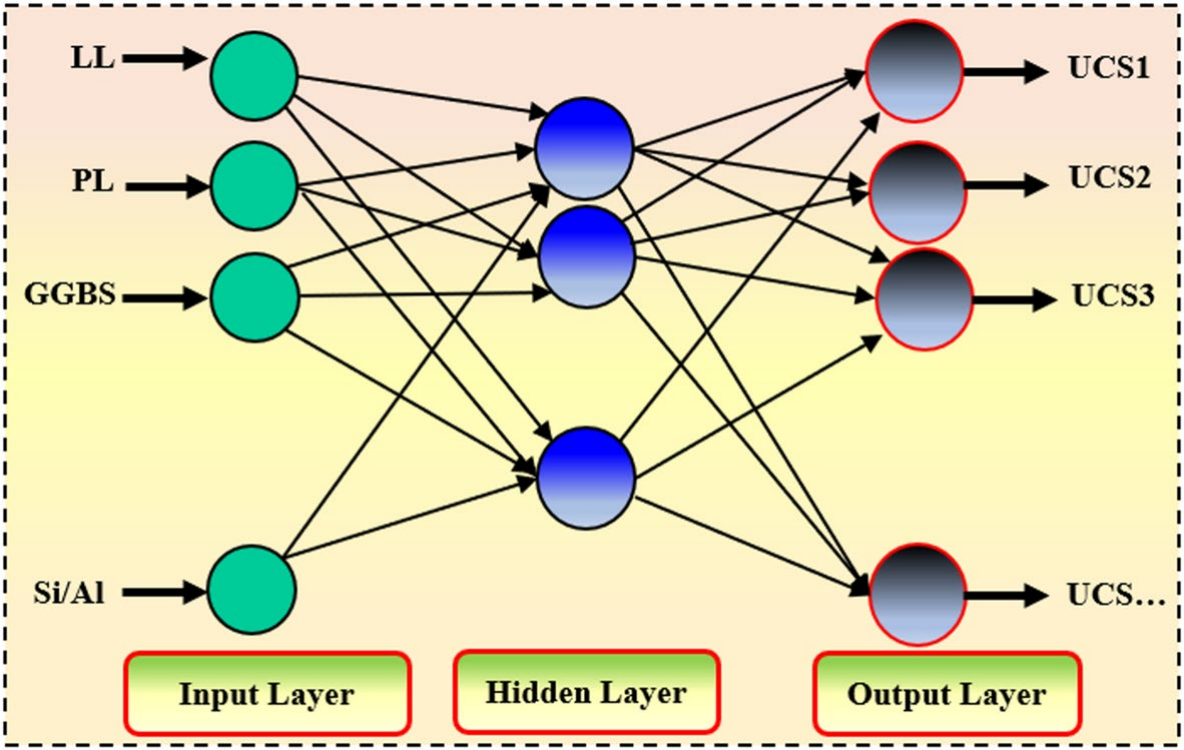
Structure of the ELM model.

Huang et al.^55^ initially created the ELM model as a quick and effective learning procedure for SLFNs (single hidden layer feedforward neural networks). In order to reach a final value, common FNNs employ slow gradient descent optimization approaches, in which the network’s parameters are gradually changed to minimize a related cost function. Long training times and, in certain situations, local minima in an optimization search space may arise from the use of data scaling or normalization. ELM, on the other hand, allows for the random assignment of input weights and biases as well as the analytical computation of output weights using the minimum-norm least squares approach. An unlimited global mining technique like this shortens training timeframes and has shown strong generalization ability^8,28,29,31,34,55–59^. It also removes the requirement for iterative tweaking of network parameters built carefully to modify weights starting with these random values. Three primary steps are involved in building the ELM model: (i) Initialization phase, which includes assigning input weights and biases to hidden nodes at random; (ii) hidden layer mapping creates the hidden layer output matrix (H); and (iii) Output weight estimation, which uses the Moore–Penrose pseudoinverse of H to calculate the output weights (α) analytically (Fig. 7). The SLFN output formulation is as follows:

1 $$\:{\mathrm{y}}_{FRS}=\:\sum\:_{j=1}^{m}{{\upalpha\:}}_{i,g}\left(\sum\:_{i=1}^{n}{w}_{i,j}{x}_{i}+{a}_{j}\right)$$

where $\:{\mathrm{y}}_{FRS}$ is the predicted output, g(.) is the activation function, $\:{w}_{i,j}$and $\:{a}_{j}$ are the randomly assigned input weights and biases, and $\:{{\upalpha\:}}_{i,g}$​ represents the output weights.

The hidden layer output matrix (H) is defined as:

2 $$\:{\mathrm{H}}_{\left({w}_{i,j,}{\:{a}_{j},x}_{i}\right)}=\:\left[\begin{array}{ccc}g\left({w}_{\mathrm{1,1}}{x}_{1}+{a}_{1}\right)&\:\dots\:&\:g\left({w}_{1,m}{x}_{1}+{a}_{m}\right)\\\:\vdots&\:\ddots\:&\:\vdots\\\:g\left({w}_{n,1}{x}_{n}+{a}_{1}\right)&\:\dots\:&\:g\left({w}_{n,m}{x}_{n}+{a}_{m}\right)\end{array}\right]$$

The final prediction is obtained as:

3 $$\:y=\:H{\upalpha\:}\:\mathrm{w}\mathrm{h}\mathrm{e}\mathrm{r}\mathrm{e}\:{\upalpha\:}\:\mathrm{i}\mathrm{s}\:\mathrm{d}\mathrm{e}\mathrm{t}\mathrm{e}\mathrm{r}\mathrm{m}\mathrm{i}\mathrm{n}\mathrm{e}\mathrm{d}\:\mathrm{u}\mathrm{s}\mathrm{i}\mathrm{n}\mathrm{g}:\:{\upalpha\:}=\:{H}^{\dag}\mathrm{T}$$

Here, $\:{H}^{\dag}$is the Moore–Penrose pseudoinverse of H, and T is the target vector.

ELM features a number of activation functions, including as sinusoidal, sigmoid, and linear. ELM can distinguish hidden parameters pertaining to stochastic initialization from analytically calculated output weights, in contrast to computationally demanding gradient descent algorithms on neural networks that iteratively modify all parameters. This makes the implementation computationally cheap and helps with quick convergence.

#### Fruit fly algorithm and its enhancement

**Fig. 8:**
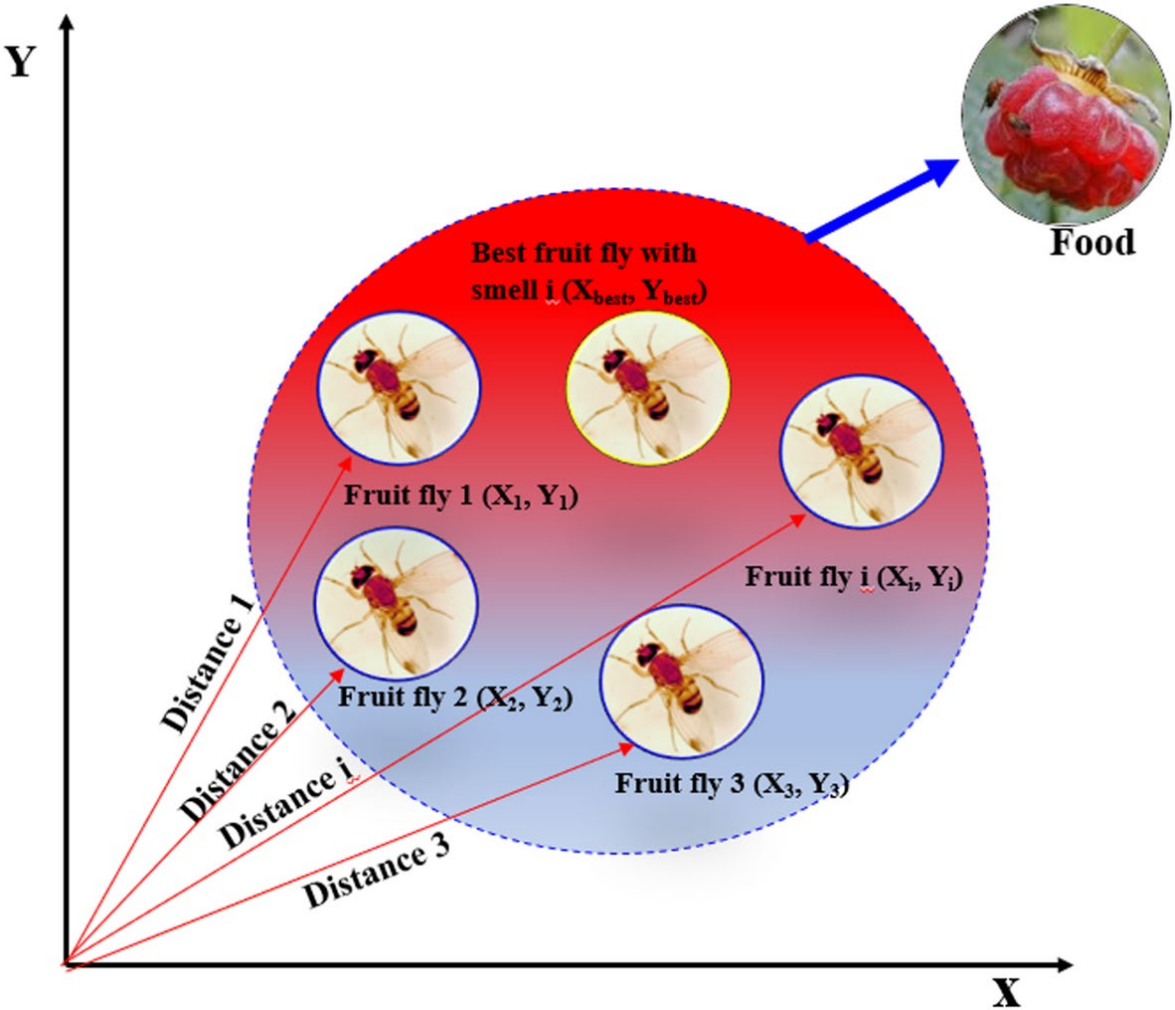
The process of food searching iterative of a fruit fly swarm.

The fruit fly optimization algorithm (FOA)^54^ is also called foraging algorithm because it is based on the foraging behavior of a Drosophila, which has a great aptitude for olfactory and visual senses. A fruit fly can smell the food from 40 km and, when it is close enough to the food source, uses its visual sense to actually locate the food and move towards it. Each iteration, the best performing fruit fly shares its information with the swarm, and all the remaining fruit flies are able to base the next search on the best-known information. Figure 8 depicts the entire foraging process of the fruit fly population.

The FOA is a global optimization algorithm derived from the food-foraging behavior of the fruit flies^60^. The algorithm replicates the acute olfaction and vision of fruit flies, enabling an iterative population-based search of the solutions space of an optimization problem. FOA has low computational complexity, a simple structure, is random, and has fast convergence and is well-suited to optimizing quantities to a global solution^60,61^. The steps for FOA are listed below:

**Step 1**: **Initialization of the fruit fly population**: Initialize the fruit fly population with a specified population size N, maximum iteration count T_max_ ​, and initial population location (X_axis_, Y_axis_) for each fruit fly i:

4 $$\:{\mathrm{I}\mathrm{n}\mathrm{i}\mathrm{t}\mathrm{i}\mathrm{a}\mathrm{l}\mathrm{i}\mathrm{z}\mathrm{a}\mathrm{t}\mathrm{i}\mathrm{o}\mathrm{n}\:\mathrm{X}\_}_{axis}\:;$$

5 $$\:{\mathrm{I}\mathrm{n}\mathrm{i}\mathrm{t}\mathrm{i}\mathrm{a}\mathrm{l}\mathrm{i}\mathrm{z}\mathrm{a}\mathrm{t}\mathrm{i}\mathrm{o}\mathrm{n}\:\mathrm{Y}\_}_{axis}\:;$$

**Step 2**: **Random search direction and distance**: Assign random direction and distance for each individual fruit fly i to search for food using its sense of smell:

6 $$\:{\mathrm{X}}_{i}=\:{\mathrm{X}\_}_{axis}+Random\:Value$$

7 $$\:{\mathrm{Y}}_{i}=\:{\mathrm{Y}\_}_{axis}+Random\:Value$$

**Step 3**: **Estimation of distance and taste concentration value** Since the precise food location is initially unknown, estimate the distance D_i_ from the origin for each fruit fly i:

8 $$\:{D}_{i}=\:\sqrt{{{x}_{i}}^{2}+{{y}_{i}}^{2}}$$

Calculate the taste concentration determination value S_i_​, defined as the reciprocal of the distance:

9 $$\:{S}_{i}=\:\frac{1}{{D}_{i}}$$

**Step 4**: **Evaluation of taste concentration**: Switch the smell concentration judgment value (S) out for the smell concentration judgment function (also called the Fitness function) in order to find the smell concentration (Smell_i_) of the individual location of the fruit fly.

10 $$\:{Smell}_{i}=\:Fuction\:\left({S}_{i}\right)$$

**Step 5**: **Identification of optimal individual**: Define the fruit fly with the highest taste concentration Smell.

11 $$\:\left[best\:Smellbest\:index\right]=\:min\:\left(Smell\right)$$

**Step 6**: **Update population position**: The individual’s coordinates are kept with the ideal flavor concentration value. The colony of flies will then fly to this location by utilizing its visual advantage:

12 $$\:\left[Smellbest\right]=\:bestSmell,$$

13 $$\:{X\_}_{axis}=\:X\:bestIndex,$$

14 $$\:{Y\_}_{axis}=\:Y\:bestIndex,$$

**Step 7**: **Termination check**: Assess whether the maximum iteration count T_max_ or target accuracy has been achieved. If not, repeat *Steps 3–6*; otherwise, return the optimal fruit fly individual as the solution.

#### Enhancement of the FOA

The traditional version of this approach includes a fixed search radius and involves a random search in all directions, which could jeopardize its stability and search accuracy^62,63^. To solve these complications so that the algorithm can be more stable and produce more accurate results we propose a new search mechanism. Using the sine and cosine functions, we will divide the search space into sectors, with each sector’s center being the population coordinates where fruit flies can disperse evenly. After that, every fruit fly in that sector will take a stride of a certain size and have an equal probability of flying in any direction. This considerably reduces complications associated with the instability of varying the iteration step size helping improve the stability and convergence of the algorithm. The basic flowchart for the sector FOA (SFOA) is presented in Fig. 2. The following are the procedures involved in creating individual fruit flies using the sector search mechanism:

**Step 1**: **Define the sector angle**: Calculate the sector angle based on the population size N, where r represents the angular interval of each sector:

15 $$\:r=\:0\::\:\frac{2\pi\:}{N-4}:\:2\pi\:$$

**Step 2**: **Generate fruit fly individuals in sectors**: Use the sine and cosine functions to randomly place fruit fly individuals within each sector, centered on the population coordinates. In this case, L is the search radius, and R is a random value between 0 and L:

16 $$\:\left\{\begin{array}{c}{\mathrm{X}}_{i}=\:{{\mathrm{X}}_{-}}_{axis}+R\:sin\left(r\right)\\\:{\mathrm{Y}}_{i}=\:{\mathrm{Y}\_}_{axis}+R\mathrm{cos}\left(r\right)\end{array}\right.,\:i<N-4$$

**Step 3**: **Apply fixed step size in key directions**: In the four essential sector directions (π/4, 3π/4, 5π/4, and 7π/4), substitute a fixed step (n ∈ {1, 3, 5, 7}) for the traditional random step size to reduce inefficient iterations. Following that, individual fruit flies are produced as follows:

17 $$\:\left\{\begin{array}{c}{\mathrm{X}}_{i}=\:{{\mathrm{X}}_{-}}_{axis}+L\:sin\left(r\right)\\\:{\mathrm{Y}}_{i}=\:{\mathrm{Y}\_}_{axis}+L\mathrm{cos}\left(r\right)\end{array}\right.,T=\frac{n\pi\:}{4},\:n=\mathrm{1,3},\mathrm{5,7}.$$

#### SFOA-ELM

**Fig. 9:**
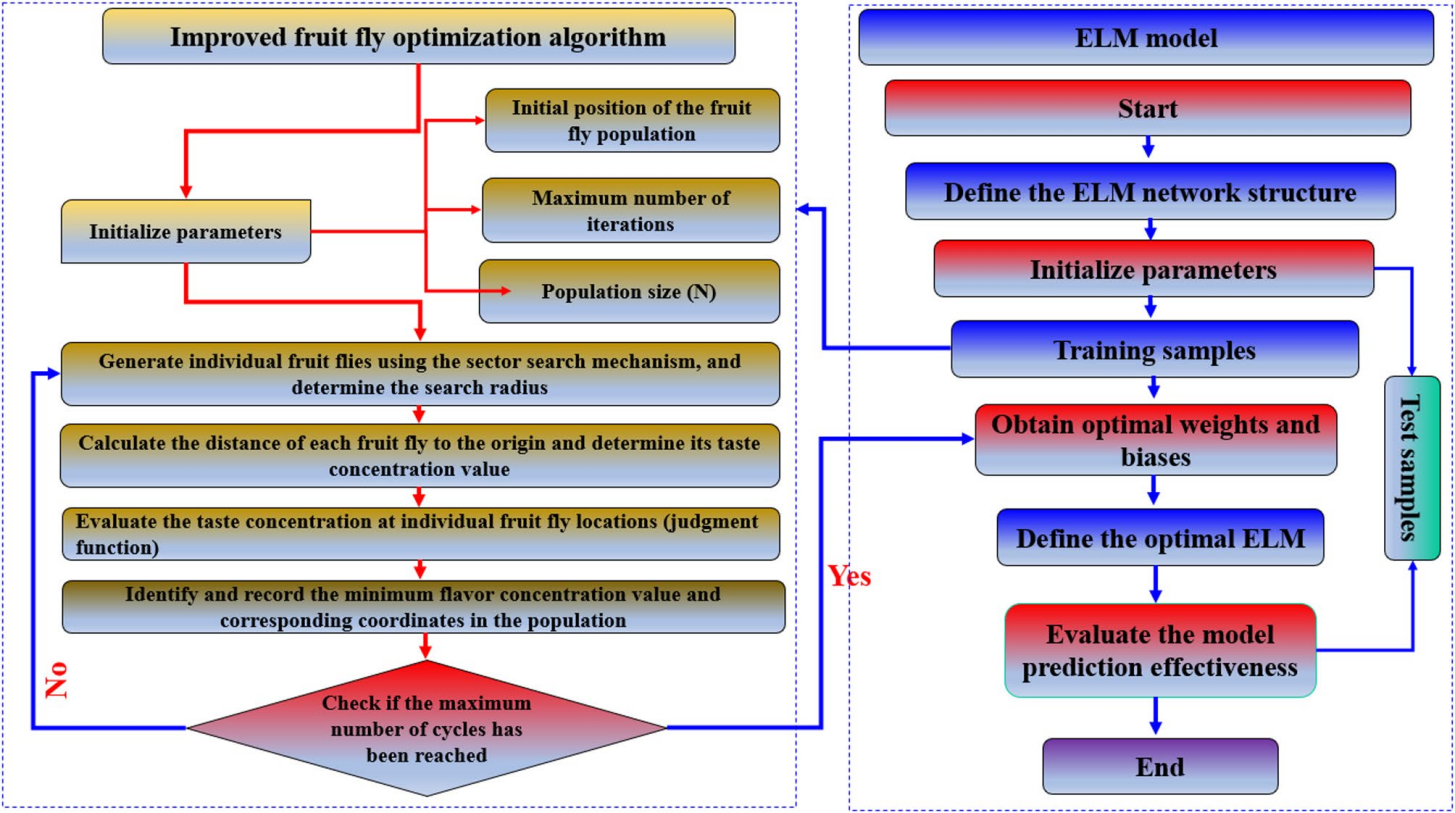
SFOA-ELM flowchart.

The ELM model’s hidden layer weights and bias values are typically and randomly initialized, which may have an impact on the model’s capacity for generalization and rate of learning. The SFOA was created in this work to optimize the weights and bias in order to overcome these problems. By doing this, the ELM’s random parameter initialization problems were resolved. The weights and bias values of the ELM are represented by each fruit fly. The flavor concentration judgment function in this instance is the MSE between the training dataset’s actual and predicted values. Depending on what happens first, the optimization process ends based on either the maximum iteration limit or the convergence criterion. Following the completion of each stage, the values that comprise the optimal ELM output were discovered. The workflow of the prediction process is depicted in Fig. 9, and the SFOA-ELM model is created to forecast consumption behavior after the SFOA identified the optimal weights and bias values.

**Step 1**: **Data preparation**: As the first sample dataset, use the data on UCS that has been gathered. Normalize the filtered data, divide it into training and test sets at an 8:2 ratio, then use correlation analysis to find important behavioral feature elements.

**Step 2**: **Population and initial setup** Define the fruit fly population size, iteration step count, and initial position, and compute the sector angle using Eqs. 4 and 5.

**Step 3**: **Sector search initialization**: Using Eqs. 15, 16, and 17, put the sector search technique into practice. Assign the ELM’s initial weights and hidden layer bias values to the fruit flies’ positional coordinates in order to generate fixed directional movements for each individual.

**Step 4**: **Distance and taste concentration calculation**: Use Eq. (11) to calculate the distance between each fruit fly’s location and the origin. Derive the taste concentration determination value S_i_ using Eq. (9).

**Step 5**: **Taste concentration evaluation**: Enter the value S_i_ for the taste concentration determination into the function that evaluates taste concentration. The flavor concentration function used in this investigation is the training set’s mean square error.

**Step 6**: **Optimal individual identification** Identify the fruit fly individual with the minimum taste concentration within the population using Eq. (10).

**Step 7**: **Position update**: Determine the optimal individual and its coordinates within the population and update the population’s position accordingly with Eqs. (11) and (12).

**Step 8**: **Iteration termination**: Verify whether the minimum error threshold or maximum iteration limit has been met. Stop the iteration and extract the ideal input weights and hidden layer bias values if you’re pleased; if not, go back to Step 3.

**Step 9**: **Model construction**: Integrate the optimized weights and hidden layer bias values into the ELM to construct the SFOA-ELM model.

**Step 10**: **Prediction and evaluation**: Input the training samples into the SFOA-ELM model to generate prediction outcomes for UCS behavior. Assess the model’s predictive performance using established evaluation metrics.

The summary comparing the three models ELM, FOA-ELM, and SFOA-ELM, Key characteristics, advantages, limitations are shown in Table 3. Additionally, Table 4 summarizes the important hyperparameters used to implement and compare the three models (ELM, FOA-ELM, and SFOA-ELM). These parameters provide equitable benchmarking, manage randomness when necessary, and preserve computational uniformity throughout experiments.

**Table 3:** Main characteristics of proposed models.

Aspect	ELM	FOA-ELM	SFOA-ELM
Description	A fast learning algorithm for SLFNs using random initialization and analytical output weight calculation.	A global optimization algorithm inspired by fruit fly foraging behavior, using olfactory and visual senses to locate solutions.	An enhanced ELM model where SFOA optimizes ELM’s hidden layer weights and biases to improve generalization and learning speed.
Optimization approach	Uses Moore-Penrose pseudoinverse for output weights (α = H⁺T), avoiding gradient descent.	Iterative population-based search with random direction and distance, optimizing via taste concentration (fitness function).	Combines SFOA’s sector-based search with ELM, using fixed step sizes and MSE as the taste concentration function to optimize weights.
Initialization	Random assignment of input weights and biases (n_hidden_neurons = 30, random_state = 42 in code).	Random initial population location (X_axis_, Y_axis_) and search direction/distance for each fruit fly.	Sector-based initialization with sine/cosine functions, fixed step sizes (n ∈ {1, 3, 5, 7}) in key directions (π/4, 3π/4, 5π/4, 7π/4).
Computational complexity	Low, as it eliminates iterative gradient descent; (n_features × n_hidden_neurons) for matrix operations.	Low complexity with simple structure, (N × Tmax) where N is population size and Tmax is iteration count.	Slightly higher than FOA due to sector calculations, but optimized for efficiency, (N × Tmax + sector overhead).
Activation functions	Supports sigmoid, tanh, relu (sigmoid used in code: 1/(1 + exp(-x))).	focuses on optimization, not neural activation.	Inherits ELM’s activation functions (e.g., sigmoid), optimized by SFOA.
Parameter Tuning	No iterative tuning; random initialization followed by pseudoinverse.	Relies on population size, iteration count, and random search parameters.	Optimizes ELM’s weights and biases using SFOA, reducing reliance on random initialization.
Limitations	Random initialization may lead to variability in performance.	Fixed search radius and random directions can reduce stability and accuracy.	Increased complexity from sector mechanism; requires tuning of sector parameters.

**Table 4:** Hyperparameters used in the models.

Parameter	ELM	FOA-ELM	SFOA-ELM	Notes / Justification
Number of hidden neurons	30	30	30	Selected after preliminary trials balancing accuracy and overfitting risk
Activation function	Sigmoid	Sigmoid	Sigmoid	Standard choice for ELM in regression tasks; g(x) = 1 / (1 + exp(-x))
Population size (N)	—	10	10	Small population to keep computation lightweight while enabling effective search
Maximum iterations	—	100	100	Sufficient for convergence as observed in MSE plots.
Random seed (initialization)	42	42	42	Ensures reproducibility of random weight/bias assignment and fruit fly positions
Number of independent runs	10	10	10	Reported metrics represent mean values over 10 runs to account for stochasticity

### Model training and evaluation metrics

#### Error estimation function and model efficiency

Different statistical parameters, including mean squared error (MSE), root mean squared error (RMSE), R^2^, relative root mean squared error (rRMSE), Mean absolute percentage error (MAPE), Mean absolute error (MAE), and Index of agreement (IA), were used to evaluate the models (Eqs. 18–21).

18 $$\:MSE=\:\frac{1}{N}\sum\:_{i=1}^{N}{({O}_{i}-{P}_{i})}^{2}$$

19 $${R^2}={\left[ {\frac{{\sum {\left( {{o_i} - \overline {o} } \right)\left( {{p_i} - \overline {p} } \right)} }}{{\sqrt {\sum {{{\left( {{o_i} - \overline {o} } \right)}^2}\sum {{{\left( {{p_i} - \overline {p} } \right)}^2}} } } }}} \right]^2}$$

20 $$RMSE=\sqrt {\frac{{\sum\limits_{1}^{n} {{{\left( {{o_i}-{p_i}} \right)}^2}} }}{N}}$$

21 $$rRMSE=100\,\left( {\frac{{RMSE}}{{\bar {O}}}} \right)$$

22 $$MAE=\frac{1}{N}\sum\limits_{{i=1}}^{N} {\left| {{o_i} - {p_i}} \right|}$$

23 $$IA=1 - \frac{{\sum\nolimits_{{i=1}}^{N} {{{\left( {{o_i} - {p_i}} \right)}^2}} }}{{\sum\nolimits_{{i=1}}^{N} {{{\left( {\left| {{o_i} - \overline {o} } \right|+\left| {{p_i} - \overline {o} } \right|} \right)}^2}} }}$$

where, o_i_ and p_i_ are the observed and the predicted values, respectively, and are the mean values of observed and the predicted values, respectively.

## Results and discussion

The computational work for this study was done on a personal laptop with an Intel dual-core 2.0 GHz processor and 8 GB of RAM, running Windows 10. Three models ELM, FOA-ELM, SFOA-ELM were created and compared in order to determine the optimal strategy. A random search optimization method was used to train the baseline ELM model. Without making any presumptions about the objective function’s structure, this approach efficiently generates and analyzes random inputs for it.

### Analysis of algorithm optimization

**Fig. 10:**
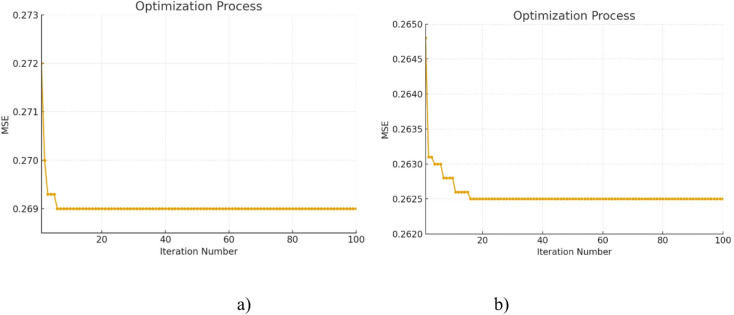
MSE convergence of **a**) FOA-ELM and **b**) SFOA-ELM.

**Fig. 11:**
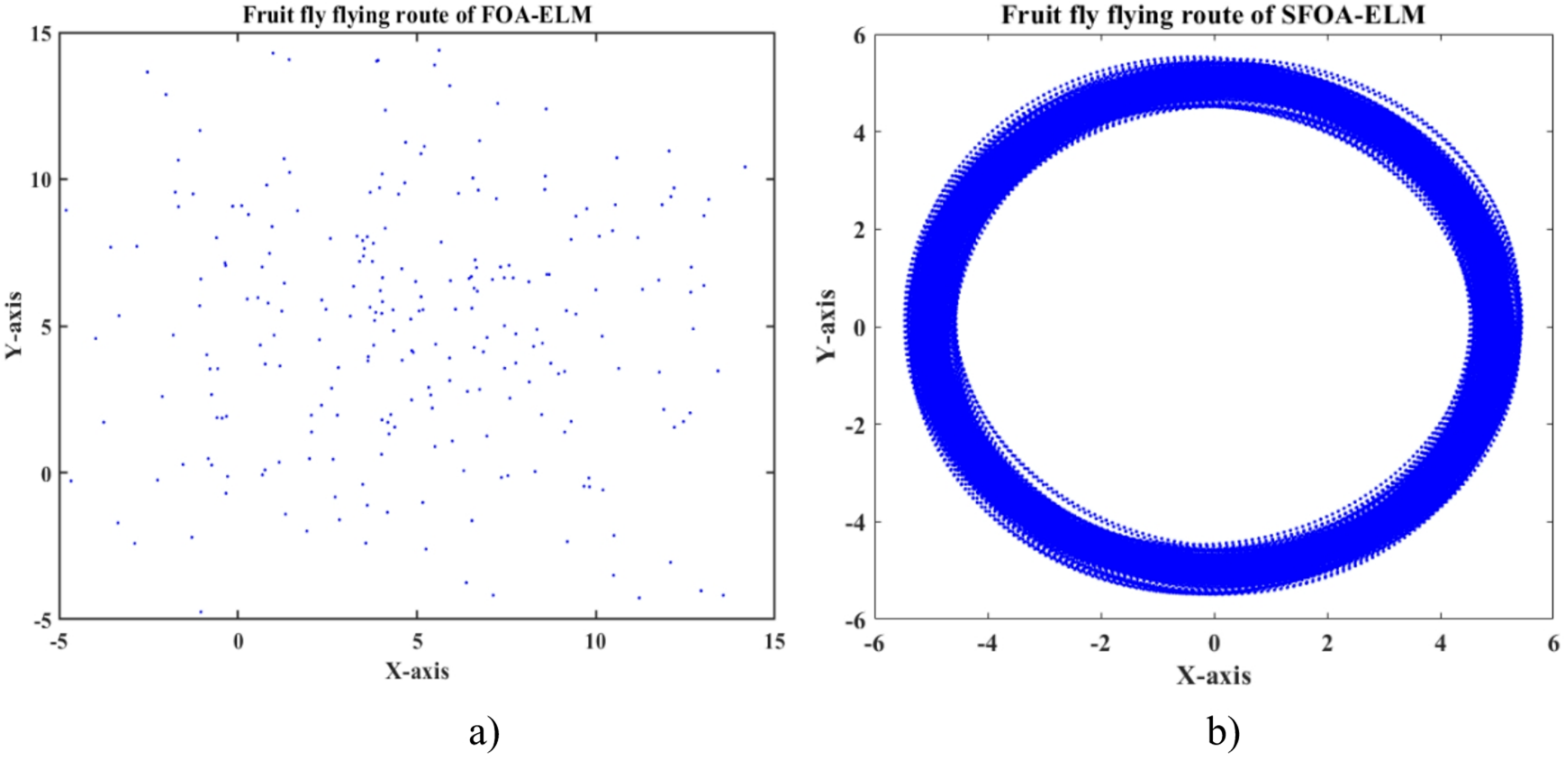
Fruit fly flying route of **a**) FOA-ELM and **b**) SFOA-ELM.

As shown in Figs. 11 and 10, there are significant differences in the optimization processes of FOA-ELM and SFOA-ELM when both methods are initialized with a fruit fly population at [0,1], a maximum number of 100 iterations, a population size of 10, and 10 hidden layer nodes in the ELM. The fruit fly flying route for FOA-ELM (Fig. 11a) shows a spread across the X-axis (0 to 15) and Y-axis (-5 to 15), suggesting that the search process is inefficient and not well organized. The random search mechanism of FOA contributes to this inefficiency and creates search directions that are not evenly distributed and less stable in their convergence^61,64,65^. The fruit fly flying route for SFOA-ELM (Fig. 11b) shows that it is more focused, and the flight directions are centered on the origin (0,0) across the X-axis (-6 to 6) and Y-axis (-6 to 6). The more structured search comes from SFOA’s process of creating a virtual circle with the population coordinates as a center and the search step as the radius to split the population into sections. Each fruit fly travels along its assigned section in an even number of steps with an even distribution and then again during the next iteration. The SFOA method is recommended for increased search efficiency as it allows the search to occur in a more uniform manner across the coordinate space without physically controlling fruit flies during the search.

In the MSE convergence context, FOA-ELM (Fig. 10a) starts from 0.273, reduced quickly to 0.269 in 20 iterations, and stabilized from then on, while SFOA-ELM (Fig. 10b) starts from 0.2650, decreases to 0.2625 quickly and stayed stable also. FOA-ELM had a slower convergence than SFOA-ELM due to its random nature with optimization strategy. On the other hand, SFOA-ELM, which has a sector-based systematic movement during the optimization process, has provided better stability and accuracy during the convergence process. Generally, in this problem, SFOA-ELM outperforms FOA-ELM which provides a lower error and better optimization process due to its organized flight path and lower MSE process.

Although the absolute performance gain of SFOA-ELM over FOA-ELM is moderate (19% reduction in testing MSE and 1.4% increase in testing R²), the improvement is consistent across all evaluation metrics (RMSE, MAE, rRMSE, and IA) and is achieved with only marginal additional computational cost. Both models employ identical population size (*N* = 10) and iteration limits (100), while the sector-based operations in SFOA introduce only O(N) complexity per iteration, which is negligible for the present problem scale. More importantly, the sector search strategy significantly enhances convergence stability. SFOA-ELM attains a lower and more consistent MSE plateau at earlier iterations (approximately 15–20) compared with FOA-ELM, which exhibits greater fluctuation. This improved robustness reduces sensitivity to random initialization and minimizes the risk of unstable runs, an important advantage for geotechnical predictive modeling, where repeated simulations and sensitivity analyses are often required.

### Comparison between the models

**Fig. 12:**
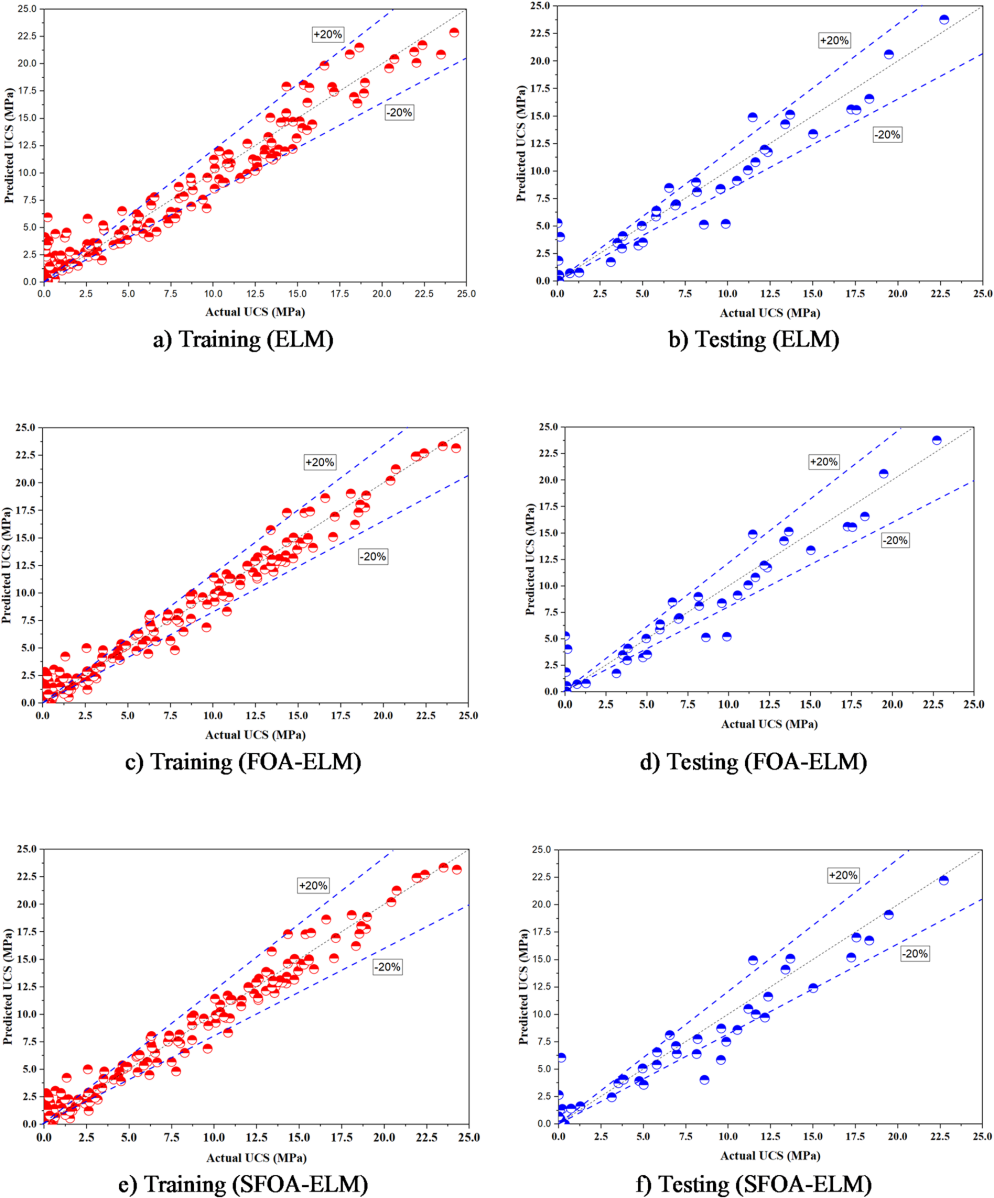
Comparison of experimental and predicted results for ML-algorithms.

**Fig. 13:**
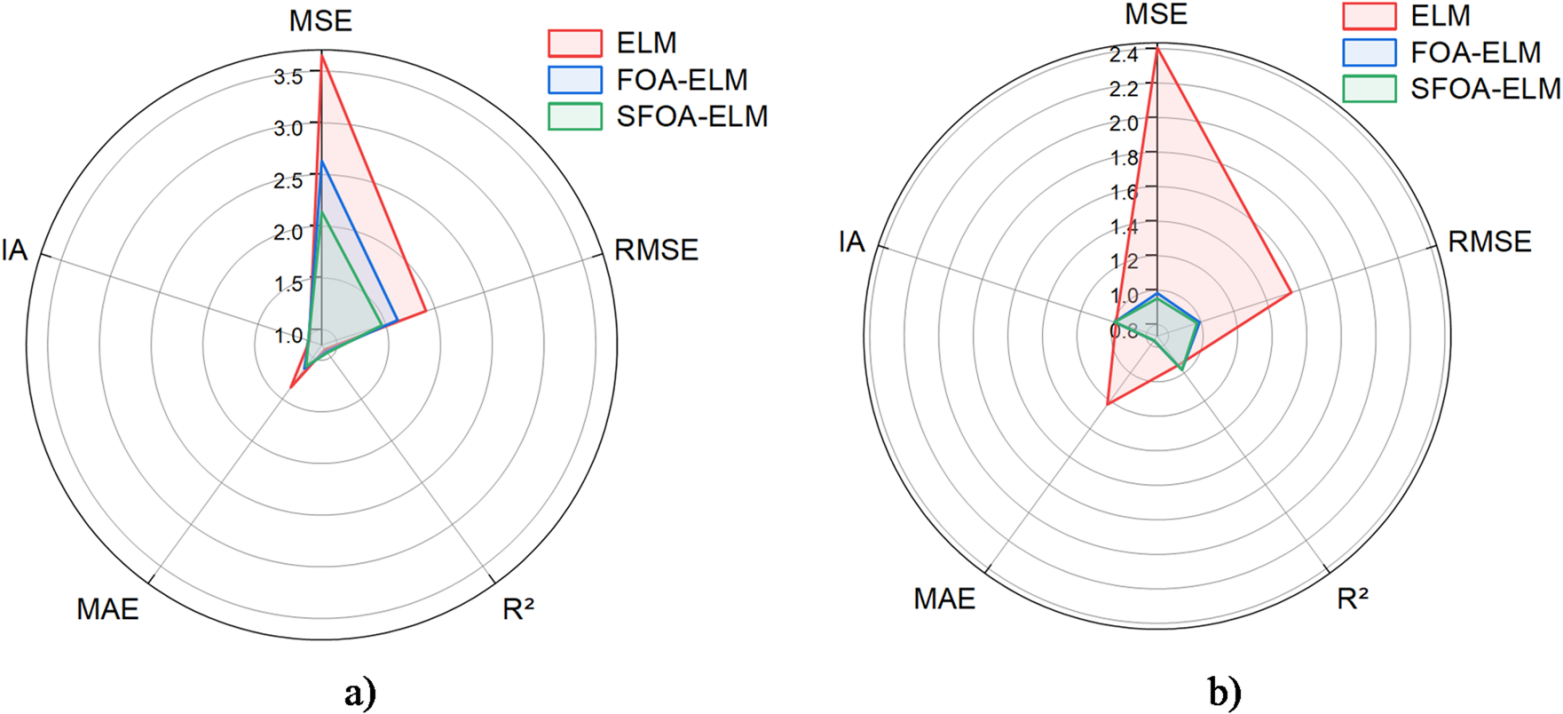
Radar charts of the proposed ML-algorithms **a**) Training set and **b**) Testing set.

The proposed ML models show observable differences in predictive capability and their overall performance is summarized in Fig. 12; Table 5. The ELM was used as a baseline to compare the performance of the other methods and achieved an R² of 0.9432 in training and 0.9104 in testing (RMSE = 1.55 MPa and 1.91 MPa in training and testing, respectively). This indicates that the ELM had moderate predictions on UCS records, but with limited robustness in the higher UCS data range. The collaborative use of FOA with ELM in FOA-ELM improved the predictive modeling, removing some error and reducing RMSE (0.99 MPa and 1.62 MPa in training and testing, respectively) and improving the R² of the derived model (R² 0.9768 and 0.9218 in training and testing, respectively). SFOA-ELM (the improved version of FOA) performed overall very well showing the lowest RMSE (0.98 MPa in training; 1.462 MPa in testing), the lowest rate of error (rRMSE = 17.27% in training; 24.25% in testing), and the highest degree of explanatory power. Visually, SFOA-ELM predictions show the densest clustering around the 1:1 line with ~ 98% of training data and ~ 95% of testing data falling within the ÷ 20% error bands (Fig. 12e-f), demonstrating the best alignment between the measured and predicted UCS values. The consistent improvements observed in SFOA-ELM (Table 5) indicates the benefits of simplified optimization that can be used to fill the gap between computational efficiency and predictive robustness. For example, SFOA-ELM provided a 42% reduction in testing MSE compared to ELM and a 19% reduction compared to FOA-ELM. SFOA-ELM also yielded the best index of agreement (IA = 0.9943 in training; 0.9864 in testing), which confirms its superior ability to replicate experimental trends. The minimal drop in R² from training to testing shows strong generalization and limited overfitting, a necessary qualification for design applications. Additionally, the radar graphs illustrated in Fig. 13 further illustrate these advances; SFOA-ELM maintains the lowest polygon area for both training and testing set among metrics including MSE, RMSE, MAE, R², and IA. In regards to the training set, SFOA-ELM has lower MSE (0.9521), RMSE (0.9758) compared to ELM and FOA-ELM metrics. In regards to the testing set, SFOA-ELM clearly outperforms, with the lowest MSE (2.1383), RMSE (1.462) along with the highest R^2^ (0.9446) and IA (0.9864), is visually confirming performance and consistency. This positive outcome implies that SFOA-ELM is more computationally efficient than FOA-ELM and is more accurate and reliable compared to both ELM and FOA-ELM giving it substantially more value in regards to UCS prediction in geotechnical design applications.

Although the incremental gain from FOA-ELM to SFOA-ELM is smaller than from ELM to FOA-ELM, it should be viewed in context: the primary innovation lies in replacing FOA’s unstable random search with a more structured yet still lightweight mechanism, yielding better generalization and lower error variance across cross-validation folds. In practical geotechnical applications, where datasets are often small/noisy and model reliability is as important as peak accuracy, these stability improvements justify the modest additional algorithmic complexity.

**Table 5:** Evaluation metrics of different methods.

Set	Metric	ELM	FOA-ELM	SFOA-ELM
Training	MSE	2.4050	0.9829	0.9521
	RMSE	1.5508	0.9914	0.9758
	R²	0.9432	0.9768	0.9775
	rRMSE (%)	27.4519	17.5495	17.2729
	MAE	1.2260	0.7679	0.7667
	IA	0.9852	0.9941	0.9943
Testing	MSE	3.6526	2.6326	2.1383
	RMSE	1.9112	1.6225	1.462
	R²	0.9054	0.9318	0.9446
	rRMSE (%)	31.7002	26.9125	24.2545
	MAE	1.3606	1.1361	1.1073
	IA	0.9758	0.9835	0.9864

**Fig. 14:**
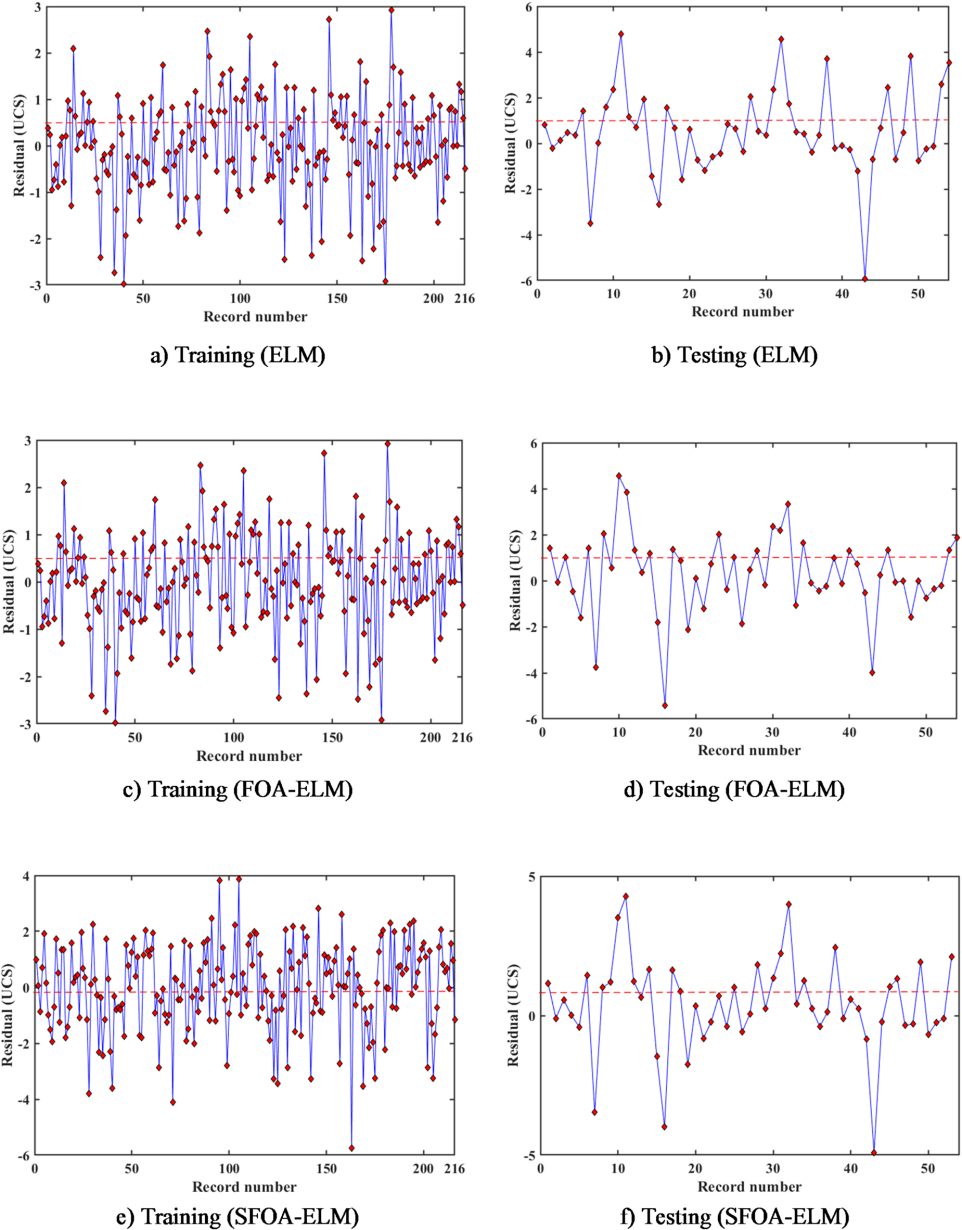
Residual plots of for ML-algorithms.

**Fig. 15:**
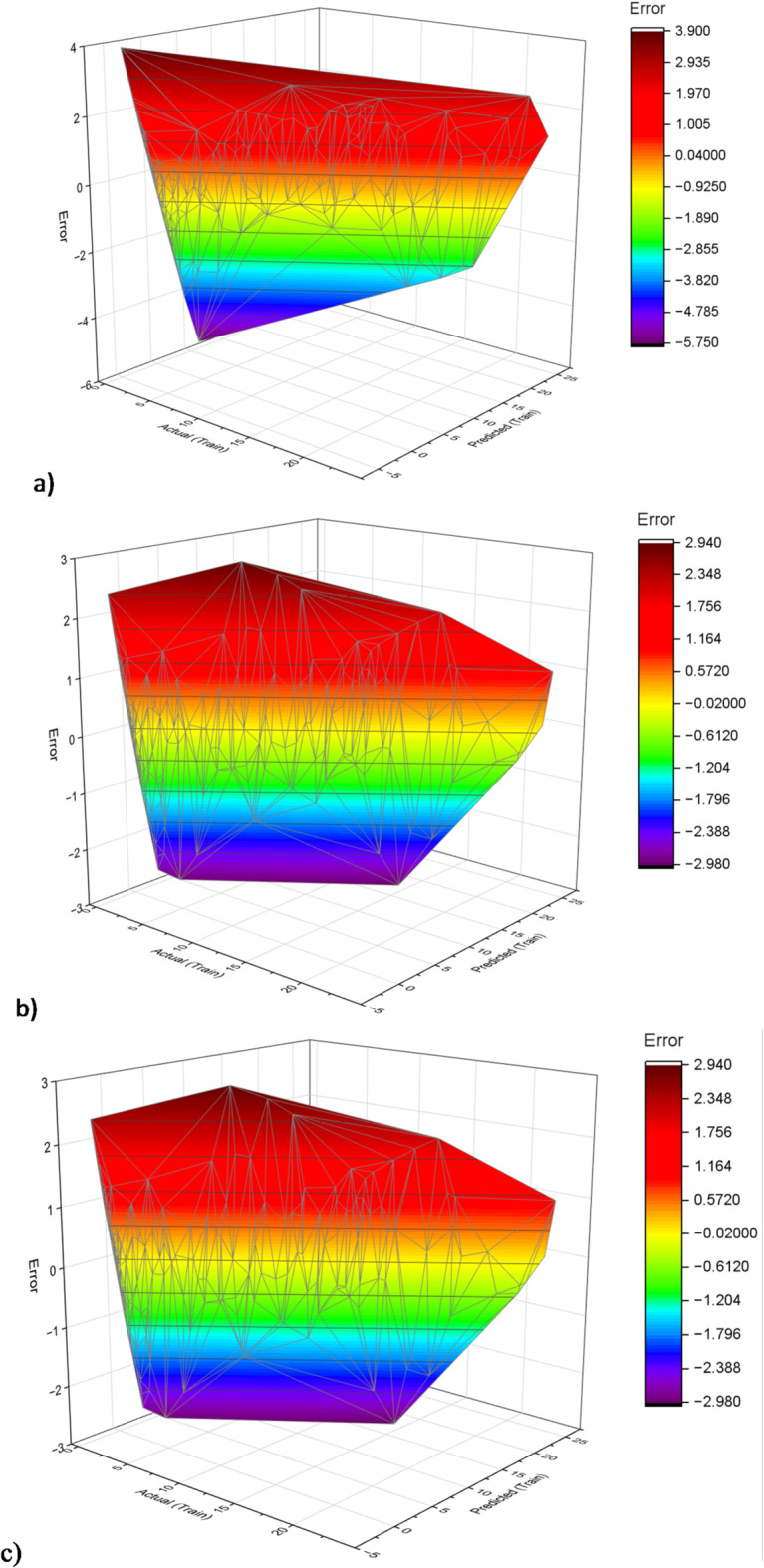
3D Error plot for training set: **a**) ELM, **b**) FOA-ELM, **c**) SFOA-ELM.

**Fig. 16:**
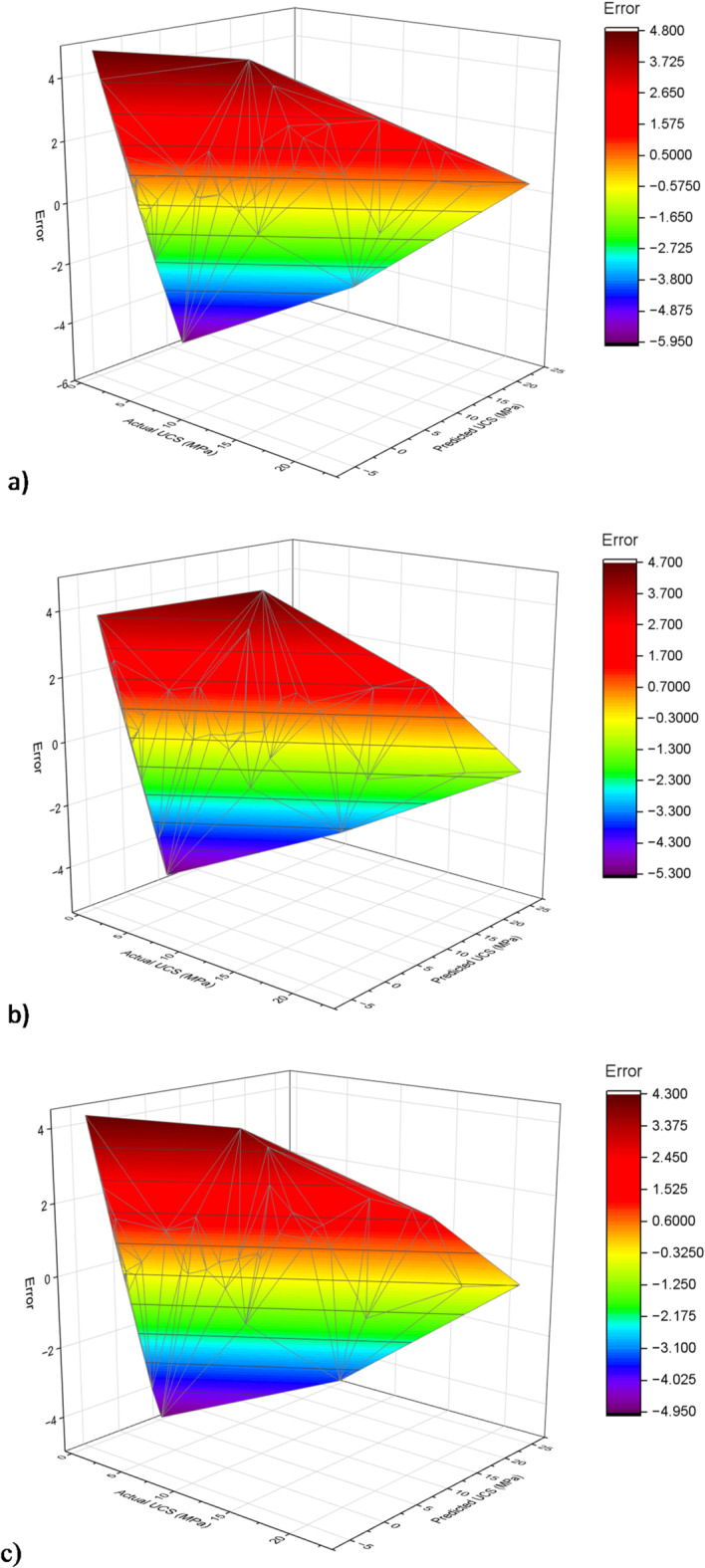
3D Error plot for testing set: **a**) ELM, **b**) FOA-ELM, **c**) SFOA-ELM.

Figure 14 shows the residual plots which provide a visual representation of the predictive accuracy of the ELM, FOA-ELM, and SFOA-ELM models across training and testing datasets, with residuals indicating the difference between predicted and actual UCS values. The ELM model clearly displays a wider spread of residuals; residuals are larger in spread particularly in the higher number of records in the training dataset, as well as a greater amount of variability in the testing dataset. This behavior is consistent with the model’s performance demonstrated earlier by RMSE of values and R^2^ values, both indicating moderate correlation with the UCS value. The FOA-ELM model, on the other hand, displays a more centered distribution with the residuals having less spread overall. This is consistent with performance metrics of RMSE values (0.99 and 1.62) and R^2^ values (0.9768 and 0.9218) indicating improved performance on the dataset overall. Similarly, SFOA-ELM model outperformed both other models yielding residuals that were the most consistently clustered around zero with a lower overall spread. This model performance is supported with a lower RMSE value (0.98 and 1.462) in training and test sets, respectively, with the highest R^2^ values. Each of these measures implies the SFOA-ELM model has superior accuracy and generalization on the dataset. The 3D error plots displayed in Figs. 15 and 16 illustrate the predictive accuracy of the ELM, FOA-ELM, and SFOA-ELM models by showing how errors are spread out in the training and test sets, respectively. For the training set (Fig. 15a), the ELM model has the widest error spread, with peaks equaling up to 3.00 MPa, suggesting major discrepancies between the predicted and actual UCS’s. The FOA-ELM (Fig. 15b) has a narrower error spread, that is largely clustered around zero, which we can attribute to its improved accuracy. The SFOA-ELM (Fig. 15c) has the smallest error spread and is seen to have the most even distribution among errors that are more closely concentrated around zero in consideration of its lowest RMSE of 0.98 MPa and highest R² of 0.9775. Likewise, for the test set, the ELM (Fig. 16a) again has the widest error spread, which peaks at 4.00 MPa, indicating a poorer degree of generalization. The FOA-ELM (Fig. 16b) also shows a tighter error distribution, and ultimately the SFOA-ELM (Fig. 16c) has the narrowest error spread amongst the three, consistent with its lower RMSE of 1.462 MPa. These 3D visualizations support the outcome of the residual plots and Table 4 indicating that SFOA-ELM consistently performed better than ELM and FOA-ELM based on precise and robust predictions in both data sets.

### Feature Importance

**Fig. 17:**
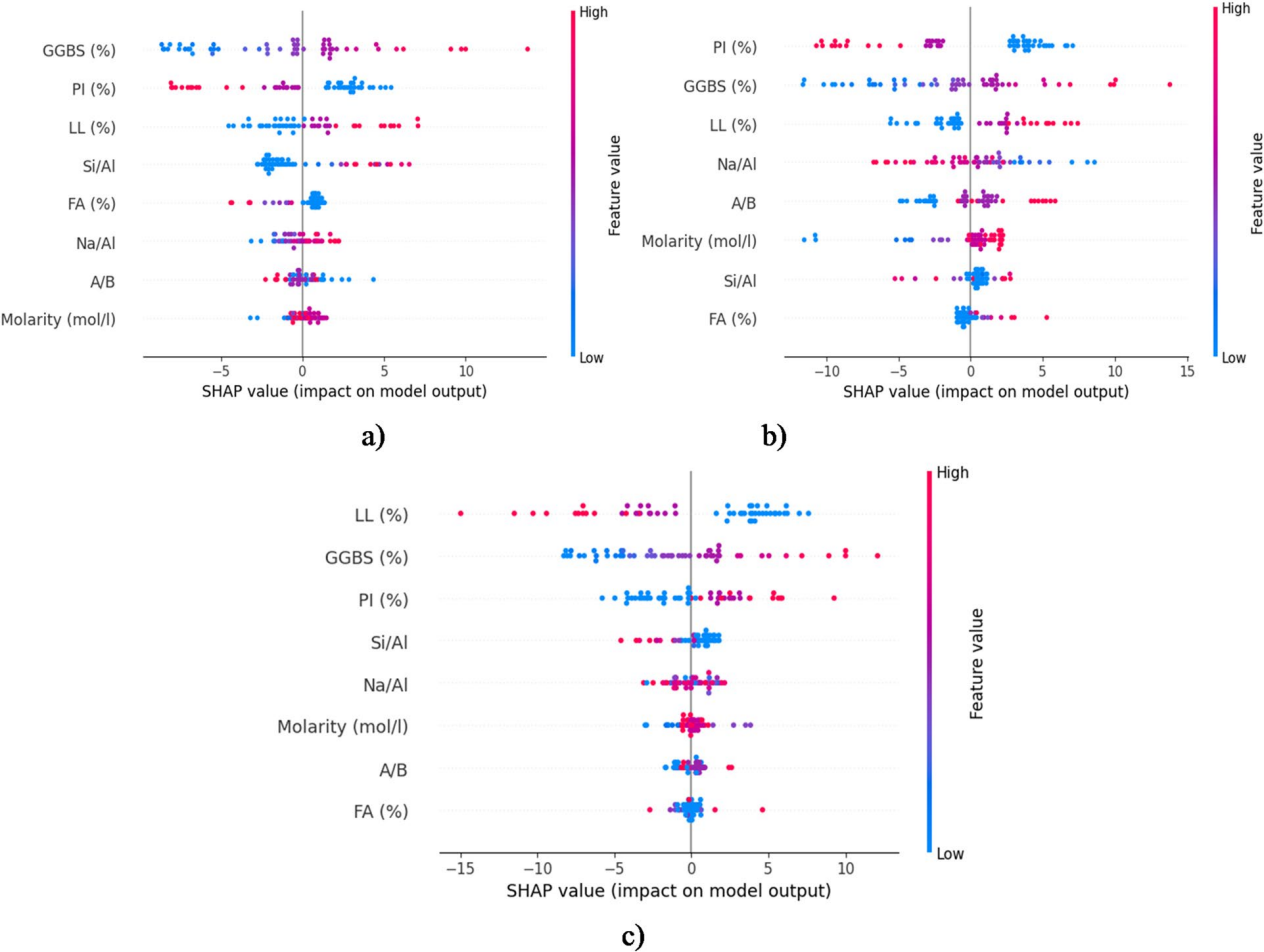
a) SHAP Value of input variables on **a**) ELM, **b**) FOA-ELM, **c**) SFOA-ELM models.

To address the interpretability limitations of previous black-box ML models for UCS prediction, SHAP analysis was employed here to provide transparent insights into feature contributions across ELM, FOA-ELM, and SFOA-ELM models. In Fig. 17, the SHAP value plots provide an understanding of the effect of input variables on the models’ predictive performance by the ELM, FOA-ELM, and SFOA-ELM. In the case of ELM (Fig. 17a), GGBS, PI, and LL had the greatest positive SHAP value, indicating that these variables positively impacted the model output, whereas Si/Al and Na/Al had moderately negative implications. This is indicative of ELM potentially relying heavily on composition (GGBS) and plasticity indices, consistent with Zhang et al.^14^, who confirmed the prevalence of binder content in UCS prediction on similar models. In the FOA-ELM (Fig. 17b), the influence also shifts, with PI, GGBS, and LL holding high positive influences, while Si/Al and Na/Al had a more robust negative consequence, which denotes variable importance as a function of optimization reflecting Abdellatief et al.^1^ findings that increased optimization can improve sensitivity of features in hybrid models. In the SFOA-ELM (Fig. 17c) there is slightly further refinement, with GGBS and PI holding strong positive commentary, while Si/Al, Na/Al, and molarity depict somewhat more balanced impacts indicating a stronger composition of parameters^32,33^. Because GGBS is a major source of calcium and reactive aluminosilicates, it facilitates the formation of C-S-H and C-A-S-H gels that cement soil particles and reduce porosity^32,33^. According to the SHAP analysis, GGBS content has the greatest positive impact on UCS. Geopolymer stabilization is especially successful in high-plasticity clays, where the three-dimensional aluminosilicate network encapsulates and bridges expansive clay minerals, reducing swelling and improving shear resistance, as further demonstrated by the significant positive contributions of PI and LL^13,33,46,66^. As demonstrated by the ideal UCS response in Fig. 17c, the moderately beneficial effect of molarity highlights the significance of sufficient alkalinity for precursor dissolution and gel polymerization.

### Effect of the combination of UCS parameters using SFOA-ELM model

**Fig. 18:**
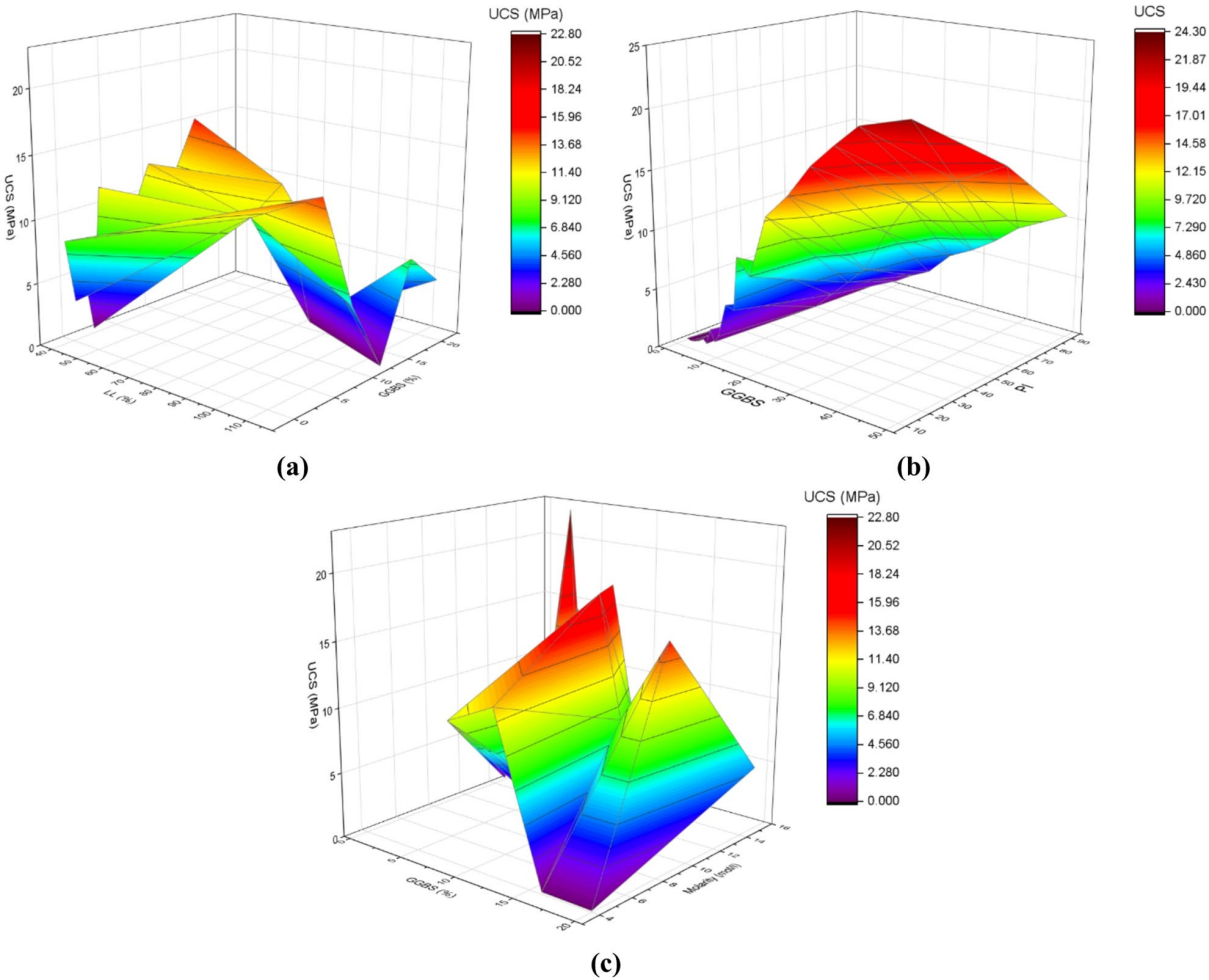
Influence of combined variable parameters on the UCS (**a**) LL vs· GGBS, (**b**) PI vs. GGBS, (**c**) GGBS vs. Molarity using the SFOA-ELM model.

The 3D plots shown in Fig. 18 demonstrate the effects of combined variable parameters on UCS via the SFOA-ELM model and the interaction effects of important geotechnical variables. Figure 18a plots LL% versus GGBS content and demonstrates a non-linear relationship with increasing UCS but an increase in peak UCS around 15.0 MPa with higher content of GGBS and relatively low LL, which is confirming a synergistic advantage. The representation of LL and GGBS, complements the work of Rezazadeh Eidgahee et al. [67]that GGBS can lead to increased UCS via stricter binder strength, when soils that have plasticity are enhanced by GGBS. In Fig. 18b in regards to PI and GGBS, UCS peaks at 20.52 MPa suggesting that higher PI values will contribute greater to the strengthening variable via GGBS, supporting Jones et al. suggestion that plasticity has significant implications on UCS. Figure 18c shows GGBS vs. the molarity with maximum UCS of 22.80 MPa with a mid-level molarity, indicating an optimal chemical structure for strength benefits that strengthens the work of Ganesh et al. [68] regarding optimal molarity can enhance the compressive strength characteristics in geopolymer systems. Thus, these most recent visualizations support the SFOA-ELM models handling of complex interactions and advanced its indicated predictive robustness seen in previous analyses. Additionally, it highlights the importance of multi parameter optimization methods in geotechnical design, from extensive evidence in reviews of previous work.

### Comparison between developed models with previously developed models

Various ML techniques have been applied to predict the UCS of geopolymer-stabilized clayey soil by overcoming the limitations of empirical and regression-based approaches that simply cannot represent complex nonlinear relationships among soil properties, binder contents, and activator parameters, as presented Table 6. Earlier studies assessed the predictive ability of ANNs and multi-variable regression (MVR)^35,40,41^; ANNs provided the best results with a testing R² = 0.982 and RMSE = 2.89 MPa compared to MVR with an R² = 0.899. Since then, newer machine learning techniques have been developed which include support vector regression (SVR) with exponential radial basis function kernels, which yield very accurate results with a testing R² = 0.994 and RMSE = 0.26 MPa; multi-gene genetic programming (MGGP) providing explicit predictive equations with R² testing results of R² = 0.960, neuro-fuzzy group method of data handling with particle swarm optimization (NF-GMDH-PSO) yielding R² = 0.903 and RMSE = 1.74 MPa, and boosting ensemble techniques such as gradient boosting (GB) giving predictive performance that’s state-of-the-art—testing R² = 0.980 and RMSE = 0.97 MPa. The present study presents a hybrid model SFOA-ELM developed utilizing a data set consisting of 270 samples that provided competitive test results of R^2^ = 0.9446, RMSE = 1.462 MPa, MAE = 1.107 MPa. Compared to the baseline ELM (R^2^= 0.9054, RMSE = 1.911 MPa), or the standard FOA-ELM (R^2^ = 0.9318, RMSE = 1.623 MPa) models, this improvement equates to a 42% reduction in testing MSE over the plain ELM. While some ensemble models such as Gradient Boosting (GB) exhibit a slightly higher R^2^ in certain configurations, the SFOA-ELM hybrid model provides an attractive balance of predictive capability, rapidness of training, efficient computational demands, and provides increased interpretability through SHAP analysis.

**Table 6:** Comparative performance of selected ML models for predicting UCS of geopolymer-stabilized clayey soils.

Ref.	Model type	Testing *R*²	Testing RMSE (MPa)	Testing MAE (MPa)	Key Advantages / Notes
Mozumder & Laskar^35^	ANN	0.982	—	—	Strong nonlinear modeling; outperformed multiple linear regression on same dataset
Mozumder et al.^40^	SVR-ERBF (best kernel)	0.994	—	—	Highest R² reported in early works using the same 270-sample dataset
Soleimani et al.^41^	MGGP	0.960	—	1.305	Provides explicit mathematical equations; superior to MLSR
Soleimani et al.^41^	ANN	0.982	—	0.083	High accuracy but black-box nature limits interpretability
Javdanian & Lee^39^	NF-GMDH-PSO	0.903	1.744	0.987	Good performance especially for UCS < 10 MPa; outperformed standard ANN
Abdullah et al.^37^	Gradient Boosting	0.980	0.969	0.585	Among the highest ensemble performances; lowest reported errors in recent comparisons
Abdullah et al.^37^	AdaBoost	0.975	1.088	0.655	Robust boosting method; slightly lower accuracy than Gradient Boosting
Current study	SFOA-ELM	0.9446	1.462	1.107	Fast training, high interpretability via SHAP, 42% MSE reduction vs. baseline ELM
	FOA-ELM	0.9318	1.623	1.136	Intermediate step showing benefit of sector enhancement
	Baseline ELM	0.9054	1.911	1.361	Extremely fast but lower accuracy without swarm optimization

## Conclusions

This research introduced and evaluated a new prediction model to predict UCS of geopolymer-stabilized clayey soils using extreme learning machine (ELM), fruit fly optimization algorithm (FOA)-based ELM, and modified sector fruit fly optimization algorithm (SFOA)-based ELM. The main conclusions are summarized in the key findings as follows:

1. The SFOA-ELM model outperformed the other methodologies, with an R² of 0.9775 during training and 0.9446 while testing, as well as the best RMSE for the training and testing datasets of 0.98 MPa and 1.462 MPa, respectively, indicating a high level of consistency and performance of the predictive model for UCS. Additionally, The SFOA-ELM improved testing MSE’s by 42% precisely, compared to the ELM, and 19% compared to FOA-ELM to reinforce the productiveness for UCS values.
2. The sector active search method of SFOA-ELM improved convergence stability, as determined by the minimize square error being reduced from 0.273 (FOA-ELM) to 0.2625 in 20 iterations, thus improving optimization performance.
3. Through SHAP analysis, it was found that GGBS, PI, and LL were the three highest positive influencers on UCS, with GGBS being the strongest correlating predictor. This finding confirms that the variable was indeed the most important factor of UCS.
4. SFOA-ELM model successfully modeled non-linear interactions, showcasing peak UCS of 22.80 MPa at GGBS vs. Molarity in respective 3D plots, which serves to illustrate the SFOA-ELM concept as an appropriate method for modeling complex relationships in geotechnical engineering studies.
5. The index of agreement (IA = 0.9864) and low rRMSE (24.25%) during testing, supports SFOA-ELM’s potential as an accurate and robust tool for geopolymer mix designs in geotechnical applications.
6. Although gradient-boosted ensembles can yield slightly higher R² in some configurations, SFOA-ELM provides an excellent trade-off between predictive performance, rapid training, low resource use, and enhanced interpretability through SHAP.

## Supplementary Information

Below is the link to the electronic supplementary material.

Supplementary Material 1

## Data Availability

- The dataset and runnable code used to develop the Sector Fruit Fly–Extreme Learning Machine (SFF-ELM) model in this study are publicly available in the Zenodo repository: DOI: ( [https://doi.org/10.5281/zenodo.19304538](https:/doi.org/10.5281/zenodo.19304538) ).- The repository contains the dataset used in the study, preprocessing scripts, and the model implementation required to reproduce the results presented in this work.

## References

[1] Abdellatief, M., Elsafi, M., Murali, G. & ElNemr, A. Comparative evaluation of hybrid machine learning models for predicting the strength of metakaolin-based geopolymer concrete enhanced with Gaussian noise augmentation. *J. Building Eng.* **111**, 113302. 10.1016/J.JOBE.2025.113302 (2025).

[2] Abdellatief, M. et al. Investigation of machine learning models in predicting compressive strength for ultra-high-performance geopolymer concrete: A comparative study. *Constr. Build. Mater.* **436**, 136884. 10.1016/J.CONBUILDMAT.2024.136884 (2024).

[3] Provis, J. L. Alkali-activated materials, Cem. *Concr Res.* **114**, 40–48. 10.1016/J.CEMCONRES.2017.02.009 (2018).

[4] Samadi, M. et al. Enhanced impact resistance of novel sustainable preplaced aggregate geopolymer concrete reinforced with steel mesh and 5D fibers. *Sci. Rep.* **15**, 29564. 10.1038/s41598-025-14281-9 (2025). 40796627 10.1038/s41598-025-14281-9 PMC12344052

[5] Murali, G., Wong, L. S., Abdulkadir, I., Algaifi, H. A. & Abdellatief, M. Sustainable transformation of waste phosphogypsum into geopolymer concrete: Comprehensive review on strength, durability, and microstructural characteristics. *J. Building Eng.* **111**, 113597. 10.1016/J.JOBE.2025.113597 (2025).

[6] Feng, Y. et al. Effects of phosphogypsum substitution on the performance of ground granulated blast furnace slag/fly ash-based alkali-activated binders. *J. Building Eng.* **70**, 106387. 10.1016/J.JOBE.2023.106387 (2023).

[7] Zhang, B. et al. Dynamic mechanical behaviour and life cycle assessment of rubberised solid waste-based geopolymer concrete. *J. Clean. Prod.* **501**, 145247. 10.1016/J.JCLEPRO.2025.145247 (2025).

[8] Ansari, S. S., Ibrahim, S. M., Hasan, S. D. & Rehman, A. U. Experiments and predictive modelling on sustainable cementitious mortar with perforated fly ash cenospheres and effectively dispersed nano silica. *Mater. Today Commun.* **47**, 113020. 10.1016/J.MTCOMM.2025.113020 (2025).

[9] Samadi, M. et al. Mechanical and mineralogical performance of sustainable cement composites with calcined palm oil leaf and calcined pine leaf Ash as supplementary cementitious materials. *Sci. Rep.* **15**, 32610. 10.1038/s41598-025-20196-2 (2025). 40968177 10.1038/s41598-025-20196-2 PMC12446477

[10] Zhang, B. et al. Effects of pretreated recycled powder substitution on mechanical properties and microstructures of alkali-activated cement. *Constr. Build. Mater.* **406**, 133360. 10.1016/J.CONBUILDMAT.2023.133360 (2023).

[11] Disu, A. A., Kolay, P. K. & A Critical Appraisal of Soil Stabilization Using Geopolymers. The Past, Present and Future. *Int. J. Geosynthetics Ground Eng.* **7**, 23. 10.1007/s40891-021-00267-w (2021).

[12] Luo, Y., Meng, J., Wang, D., Jiao, L. & Xue, G. Experimental study on mechanical properties and microstructure of metakaolin based geopolymer stabilized silty clay. *Constr. Build. Mater.* **316**, 125662. 10.1016/J.CONBUILDMAT.2021.125662 (2022).

[13] Wang, S. et al. Experimental study on material ratio and strength performance of geopolymer-improved soil. *Constr. Build. Mater.* **267**, 120469. 10.1016/J.CONBUILDMAT.2020.120469 (2021).

[14] Zhang, M., Guo, H., El-Korchi, T., Zhang, G. & Tao, M. Experimental feasibility study of geopolymer as the next-generation soil stabilizer. *Constr. Build. Mater.* **47**, 1468–1478. 10.1016/J.CONBUILDMAT.2013.06.017 (2013).

[15] Ayub, F. & Khan, S. A. An overview of geopolymer composites for stabilization of soft soils. *Constr. Build. Mater.* **404**, 133195. 10.1016/J.CONBUILDMAT.2023.133195 (2023).

[16] Zhang, M. et al. Calcium-free geopolymer as a stabilizer for sulfate-rich soils. *Appl. Clay Sci.* **108**, 199–207. 10.1016/J.CLAY.2015.02.029 (2015).

[17] Khadka, S. D., Jayawickrama, P. W., Senadheera, S. & Segvic, B. Stabilization of highly expansive soils containing sulfate using metakaolin and fly ash based geopolymer modified with lime and gypsum. *Transp. Geotechnics*. **23**, 100327. 10.1016/J.TRGEO.2020.100327 (2020).

[18] Aboulayt, A. et al. Stability of a new geopolymer grout: Rheological and mechanical performances of metakaolin-fly ash binary mixtures. *Constr. Build. Mater.* **181**, 420–436. 10.1016/J.CONBUILDMAT.2018.06.025 (2018).

[19] Yang, B. et al. Strength characteristics of modified polypropylene fiber and cement-reinforced loess. *J. Cent. South. Univ.* **24**, 560–568. 10.1007/s11771-017-3458-0 (2017).

[20] Bahmyari, H., Ajdari, M., Vakili, A. & Ahmadi, M. H. The Role of the Cement, Lime, and Natural Pozzolan Stabilizations on the Mechanical Response of a Collapsible Soil. *Transp. Infrastructure Geotechnology*. **8**, 452–472. 10.1007/s40515-020-00146-3 (2021).

[21] Salimi, M. & Ghorbani, A. Mechanical and compressibility characteristics of a soft clay stabilized by slag-based mixtures and geopolymers. *Appl. Clay Sci.* **184**, 105390. 10.1016/J.CLAY.2019.105390 (2020).

[22] Ghadir, P. & Ranjbar, N. Clayey soil stabilization using geopolymer and Portland cement. *Constr. Build. Mater.* **188**, 361–371. 10.1016/J.CONBUILDMAT.2018.07.207 (2018).

[23] Zhang, B. et al. Rubberized geopolymer concrete: Dependence of mechanical properties and freeze-thaw resistance on replacement ratio of crumb rubber. *Constr. Build. Mater.* **310**, 125248. 10.1016/J.CONBUILDMAT.2021.125248 (2021).

[24] Feng, Y. et al. Improving geopolymer concrete performance with hazardous solid waste phosphogypsum. *J. Building Eng.* **95**, 110333. 10.1016/J.JOBE.2024.110333 (2024).

[25] Chen, F. et al. An investigation of the durability of ultra-lightweight high-strength geopolymeric composites. *J. Building Eng.* **80**, 107990. 10.1016/J.JOBE.2023.107990 (2023).

[26] Abed, M. H., Abbas, I. S., Mohmmad, S. H., Saygili, A. & Agha, A. A. Performance of Soils Stabilized with Eco-friendly Mechanochemical Geopolymeric Activators. *Geotech. Geol. Eng.* **43**, 117. 10.1007/s10706-025-03073-7 (2025).

[27] Thapa, I. et al. Sustainable approach of strength measurement for soil’s stabilized with geo-polymer with hybrid ensemble models. *Results Eng.* **27**, 106133. 10.1016/J.RINENG.2025.106133 (2025).

[28] Shamim, A. S., Muhammad, I. S. & Danish, H. S. Interpretable Machine-Learning Models to Predict the Flexural Strength of Fiber-Reinforced SCM-Blended Concrete Composites. *J. Struct. Des. Constr. Pract.* **30**, 04024113. 10.1061/JSDCCC.SCENG-1496 (2025).

[29] Ansari, S. S., Ansari, M. A., Shariq, M., Mahdi, F. & Ibrahim, S. M. Ensemble Machine Learning Models to Predict the Compressive Strength and Ultrasonic Pulse Velocity of Sustainable Concrete, in: (eds Menon, N. V. C., Kolathayar, S., Rodrigues, H. & Sreekeshava, K. S.) Recent Advances in Civil Engineering for Sustainable Communities, Springer Nature Singapore, Singapore, : 57–69. (2024).

[30] Ansari, S. S., Ansari, M. A., Saqib, M., Ghazi, M. S. & Ibrahim, S. M. Impact of thermal loads on silica fume-modified lightweight concrete: Machine learning approach to assess compressive strength evolution. *Mater. Today Proc.*10.1016/J.MATPR.2024.04.054 (2024).

[31] Ibrahim, S. M., Ansari, S. S. & Hasan, S. D. Towards white box modeling of compressive strength of sustainable ternary cement concrete using explainable artificial intelligence (XAI). *Appl. Soft Comput.* **149**, 110997. 10.1016/J.ASOC.2023.110997 (2023).

[32] Azfar Shaida, M. et al. Prediction of CO2 uptake in bio-waste based porous carbons using model agnostic explainable artificial intelligence. *Fuel* **380**, 133183. 10.1016/J.FUEL.2024.133183 (2025).

[33] Mohammad, T., Ibrahim, S. M., Ansari, S. S. & Rehman, A. U. Feasibility study and optimization of limestone calcined clay composites for compressive strength using multi-layered explainable artificial intelligence models. *Mater. Today Commun.* **40**, 109676. 10.1016/J.MTCOMM.2024.109676 (2024).

[34] Abdellatief, M., Hamla, W. & Hamouda, H. AI driven prediction of early age compressive strength in ultra high performance fiber reinforced concrete. *Sci. Rep.* **15**, 20316. 10.1038/s41598-025-06725-z (2025). 40571697 10.1038/s41598-025-06725-z PMC12202810

[35] Mozumder, R. A. & Laskar, A. I. Prediction of unconfined compressive strength of geopolymer stabilized clayey soil using Artificial Neural Network. *Comput. Geotech.* **69**, 291–300. 10.1016/J.COMPGEO.2015.05.021 (2015).

[36] Tabarsa, A., Latifi, N., Osouli, A. & Bagheri, Y. Unconfined compressive strength prediction of soils stabilized using artificial neural networks and support vector machines. *Front. Struct. Civil Eng.* **15**, 520–536. 10.1007/s11709-021-0689-9 (2021).

[37] Abdullah, G. M. S. et al. Boosting-based ensemble machine learning models for predicting unconfined compressive strength of geopolymer stabilized clayey soil. *Sci. Rep.* **14**, 2323. 10.1038/s41598-024-52825-7 (2024). 38282061 10.1038/s41598-024-52825-7 PMC10822860

[38] Zhang, J., Choi, C. E., Liang, Z. & Li, R. A generic framework for mix design of geopolymer for soil stabilization: Composition-informed machine learning model. *Comput. Geotech.* **170**, 106322. 10.1016/J.COMPGEO.2024.106322 (2024).

[39] Javdanian, H. & Lee, S. Evaluating unconfined compressive strength of cohesive soils stabilized with geopolymer: a computational intelligence approach. *Eng. Comput.* **35**, 191–199. 10.1007/s00366-018-0592-8 (2019).

[40] Mozumder, R. A., Laskar, A. I. & Hussain, M. Empirical approach for strength prediction of geopolymer stabilized clayey soil using support vector machines. *Constr. Build. Mater.* **132**, 412–424. 10.1016/J.CONBUILDMAT.2016.12.012 (2017).

[41] Soleimani, S., Rajaei, S., Jiao, P., Sabz, A. & Soheilinia, S. New prediction models for unconfined compressive strength of geopolymer stabilized soil using multi-gen genetic programming. *Measurement* **113**, 99–107. 10.1016/J.MEASUREMENT.2017.08.043 (2018).

[42] Zeini, H. A. et al. Random Forest Algorithm for the Strength Prediction of Geopolymer Stabilized Clayey Soil. *Sustainability* **15**10.3390/su15021408 (2023).

[43] Ghadir, P. et al. Shear strength and life cycle assessment of volcanic ash-based geopolymer and cement stabilized soil: A comparative study. *Transp. Geotechnics*. **31**, 100639. 10.1016/J.TRGEO.2021.100639 (2021).

[44] Miraki, H. et al. Clayey soil stabilization using alkali-activated volcanic ash and slag. *J. Rock Mech. Geotech. Eng.* **14**, 576–591. 10.1016/J.JRMGE.2021.08.012 (2022).

[45] Singhi, B., Laskar, A. I. & Ahmed, M. A. Investigation on Soil–Geopolymer with Slag, Fly Ash and Their Blending. *Arab. J. Sci. Eng.* **41**, 393–400. 10.1007/s13369-015-1677-y (2016).

[46] Abdullah, H. H., Shahin, M. A. & Walske, M. L. Geo-mechanical behavior of clay soils stabilized at ambient temperature with fly-ash geopolymer-incorporated granulated slag. *Soils Found.* **59**, 1906–1920. 10.1016/J.SANDF.2019.08.005 (2019).

[47] Yan-Jun, D., Bo-Wei, Y., Kai, L. & Ning-Jun, J. Physical, Hydraulic, and Mechanical Properties of Clayey Soil Stabilized by Lightweight Alkali-Activated Slag Geopolymer. *J. Mater. Civ. Eng.* **29**, 04016217. 10.1061/(ASCE)MT.1943-5533.0001743 (2017).

[48] Rezazadeh Eidgahee, D., Rafiean, A. H., Haddad, A. & Approaches A Novel Formulation for the Compressive Strength of IBP-Based Geopolymer Stabilized Clayey Soils Using ANN and GMDH-NN *Iran. J. Sci. Technol. Trans. Civil Eng.* **44** 219–229. 10.1007/s40996-019-00263-1. (2020).

[49] Samuel, R., Puppala, A. J. & Radovic, M. Sustainability Benefits Assessment of Metakaolin-Based Geopolymer Treatment of High Plasticity Clay. *Sustainability* **12**10.3390/su122410495 (2020).

[50] Shi, X. et al. Experimental Study on the Mechanical Properties and Microstructure of Metakaolin-Based Geopolymer Modified Clay. *Molecules* **27**10.3390/molecules27154805 (2022). 10.3390/molecules27154805 PMC9369488 35956771

[51] Fakhrabadi, A., Ghadakpour, M., Choobbasti, A. J. & Kutanaei, S. S. Evaluating the durability, microstructure and mechanical properties of a clayey-sandy soil stabilized with copper slag-based geopolymer against wetting-drying cycles. *Bull. Eng. Geol. Environ.* **80**, 5031–5051. 10.1007/s10064-021-02228-z (2021).

[52] Khasib, I. A., Daud, N. N. N. & Nasir, N. A. M. Strength Development and Microstructural Behavior of Soils Stabilized with Palm Oil Fuel Ash (POFA)-Based Geopolymer. *Appl. Sci.* **11**10.3390/app11083572 (2021).

[53] Abdeldjouad, L., Asadi, A., Ball, R. J., Nahazanan, H. & Huat, B. B. K. Application of alkali-activated palm oil fuel ash reinforced with glass fibers in soil stabilization. *Soils Found.* **59**, 1552–1561. 10.1016/J.SANDF.2019.07.008 (2019).

[54] Li, B., Liao, M., Yuan, J. & Zhang, J. Green consumption behavior prediction based on fan-shaped search mechanism fruit fly algorithm optimized neural network. *J. Retailing Consumer Serv.* **75**, 103471. 10.1016/J.JRETCONSER.2023.103471 (2023).

[55] Bin Huang, G., Zhu, Q. Y. & Siew, C. K. Extreme learning machine: Theory and applications. *Neurocomputing* **70**, 489–501. 10.1016/J.NEUCOM.2005.12.126 (2006).

[56] Shariati, M. et al. A novel hybrid extreme learning machine–grey wolf optimizer (ELM-GWO) model to predict compressive strength of concrete with partial replacements for cement. *Eng. Comput.* **38**, 757–779. 10.1007/s00366-020-01081-0 (2022).

[57] Katebi, J., Shoaei-parchin, M., Shariati, M., Trung, N. T. & Khorami, M. Developed comparative analysis of metaheuristic optimization algorithms for optimal active control of structures. *Eng. Comput.* **36**, 1539–1558. 10.1007/s00366-019-00780-7 (2020).

[58] Kumar Dash, P., Kumar Parhi, S., Kumar Patro, S. & Panigrahi, R. Efficient machine learning algorithm with enhanced cat swarm optimization for prediction of compressive strength of GGBS-based geopolymer concrete at elevated temperature. *Constr. Build. Mater.* **400**, 132814. 10.1016/J.CONBUILDMAT.2023.132814 (2023).

[59] Abdellatief, M., Abd-Elmaboud, M. E., Mortagi, M. & Saqr, A. M. A convolutional neural network-based deep learning approach for predicting surface chloride concentration of concrete in marine tidal zones. *Sci. Rep.* **15**, 27611. 10.1038/s41598-025-12035-1 (2025). 40730630 10.1038/s41598-025-12035-1 PMC12307714

[60] Pan, W. T. A new Fruit Fly Optimization Algorithm: Taking the financial distress model as an example. *Knowl. Based Syst.* **26**, 69–74. 10.1016/J.KNOSYS.2011.07.001 (2012).

[61] Yin, B., Yang, J. & Li, Y. Fruit Fly Optimization Algorithm with Evolution Strategy for Magnetotelluric Data Inversion. *J. Math.* **2023**, 8810401. 10.1155/2023/8810401 (2023).

[62] Han, S. Z., Pan, W. T., Zhou, Y. Y. & Liu, Z. L. Construct the prediction model for China agricultural output value based on the optimization neural network of fruit fly optimization algorithm. *Future Generation Comput. Syst.* **86**, 663–669. 10.1016/J.FUTURE.2018.04.058 (2018).

[63] Cao, G. & Wu, L. Support vector regression with fruit fly optimization algorithm for seasonal electricity consumption forecasting. *Energy* **115**, 734–745. 10.1016/J.ENERGY.2016.09.065 (2016).

[64] Jiang, F., Zhang, W. & Peng, Z. Multivariate Adaptive Step Fruit Fly Optimization Algorithm Optimized Generalized Regression Neural Network for Short-Term Power Load Forecasting. *Front. Environ. Sci. Volume*. **10-2022**10.3389/fenvs.2022.873939 (2022).

[65] Guo, X., Zhang, X. & Wang, L. Fruit Fly Optimization Algorithm Based on Single-Gene Mutation for High-Dimensional Unconstrained Optimization Problems. *Math. Probl. Eng.* **2020**, 9676279. 10.1155/2020/9676279 (2020).

[66] Ganesh, A. C. et al. Development of alkali activated paver blocks for medium traffic conditions using industrial wastes and prediction of compressive strength using random forest algorithm. *Sci. Rep.* **13**, 15152. 10.1038/s41598-023-42318-4 (2023). 37704735 10.1038/s41598-023-42318-4 PMC10500016

